# The USP19-DnaJC7 Axis Stabilizes p53 in Cisplatin-Treated Epithelial Ovarian Cancer

**DOI:** 10.3390/cells15100925

**Published:** 2026-05-18

**Authors:** Yosuk Min, Donghyeon Kim, Hong-Beom Park, Hae-Seul Choi, Sohyun Hwang, Kwang-Hyun Baek

**Affiliations:** 1 Department of Life Sciences, CHA University, Seongnam 13488, Gyeonggi-Do, Republic of Korea; minga06@naver.com (Y.M.); ehdgus6196@naver.com (D.K.); hschoi@chauniv.ac.kr (H.-S.C.); 2 Department of Biomedical Science, CHA University, Seongnam 13488, Gyeonggi-Do, Republic of Korea; hongbeom5567@naver.com; 3 Department of Pathology, CHA Bundang Medical Center, CHA University School of Medicine, Seongnam 13520, Gyeonggi-Do, Republic of Korea; 4 Department of Bioconvergence, CHA University, Seongnam 13488, Gyeonggi-Do, Republic of Korea

**Keywords:** apoptosis, cisplatin, deubiquitinating enzyme, epithelial ovarian cancer, p53

## Abstract

Epithelial ovarian cancer (EOC) poses a challenge owing to its high rate of recurrence and drug resistance, resulting in a 5-year survival rate of 30% in advanced stages. To elucidate the molecular mechanisms underlying EOC recurrence, we analyzed transcriptome data from patients with EOC and identified elevated *USP19*, a deubiquitinating enzyme, as being elevated in patients without recurrence in our previous study. Single-cell RNA sequencing analysis revealed that increased *USP19* expression in epithelial cells is associated with activation of apoptotic pathways, suggesting that *USP19* may inhibit EOC recurrence through deubiquitination of its binding proteins. Using the protein–protein interaction database, we identified DnaJC7 as a binding partner of USP19 and confirmed their interaction experimentally. USP19-mediated deubiquitination of DnaJC7 increases its protein stability. Notably, upregulation of USP19 and DnaJC7 disrupted the interaction between p53 and MDM2, and knockdown of USP19 and DnaJC7 resulted in decreased p53 expression following cisplatin treatment. These findings highlight the therapeutic potential of enhancing the USP19-DnaJC7 axis to stabilize p53 and improve cisplatin efficacy. Promoting USP19-mediated deubiquitination by stabilizing DnaJC7 may offer a novel combination strategy to enhance the efficacy of cisplatin-based cancer therapy.

## 1. Introduction

Epithelial ovarian cancer (EOC) ranks as the seventh most common cancer among women worldwide. Owing to the lack of early symptoms or diagnostic markers, most patients are diagnosed at advanced stages (III or IV), resulting in a poor 5-year survival rate [1]. Current therapeutic approaches for EOC include targeted therapy and chemotherapy with platinum-based (cisplatin, carboplatin, and oxaliplatin) and taxane-based drugs, constituting standard treatment protocols after surgery [2]. Among these, cisplatin induces cell death through mechanisms such as DNA damage and endoplasmic reticulum (ER) stress mediated by reactive oxygen species [3,4]. Despite initial responses, many patients with EOC experience recurrence and develop resistance to platinum-based therapies, contributing to the low median 5-year survival rate observed in this population [5]. To better understand the molecular characteristics of recurrent EOC, we have investigated the EOC transcriptome data from The Cancer Genome Atlas, Gene Expression Omnibus, and our patients, identifying that patients with high expression levels of *ubiquitin-specific protease 19* (*USP19*) have a high 5-year survival rate [6]. USP19, a deubiquitinating enzyme, removes polyubiquitin chains from substrate proteins to prevent their proteasomal degradation, and may contribute to the suppression of EOC recurrence through stabilization of key target proteins [7].

Ubiquitination, a key post-translational modification, involves the attachment of ubiquitin molecules to substrates [8]. Ubiquitin can form polyubiquitin chains by conjugating to the K6, K11, K27, K29, K33, K48, K63, and M1 residues of ubiquitin, and its function depends on the amino acid residue of the polyubiquitin chain formed [9]. Ubiquitin is attached to the substrate proteins via a cascade involving E1 ubiquitin-activating enzymes, E2 ubiquitin-conjugating enzymes, and E3 ubiquitin ligases [10]. The ubiquitin-proteasome system (UPS) regulates substrate stability and degradation, with deubiquitinating enzyme (DUB) reversing the process by cleaving ubiquitin chains and stabilizing substrates [11].

USP19 belongs to the ubiquitin-specific protease (USP) family within the DUB superfamily and has been implicated in the regulation of Beclin-1, Baculoviral IAP repeat containing 5 (survivin), Coronin 2A, NIMA related kinase 9 (NEK9), Cellular Myelocytomatosis (c-Myc), Programmed cell death 1 ligand 1 (PD-L1), and other proteins [12,13,14,15,16,17]. Although the role of USP19 in cancer progression has been predominantly studied as promoting oncogenesis, studies in EOC and renal cell carcinoma suggest that higher expression levels of *USP19* correlate with improved patient survival [6,18,19,20]. Therefore, we investigated the molecular mechanisms underlying the role of USP19 in recurrent EOC and identified potential binding partners through protein–protein interaction (PPI) database.

As a candidate substrate of USP19, we selected DnaJ homolog subfamily C member 7 (DnaJC7), which stabilizes tumor suppressor protein p53 by disrupting its interaction with murine double minute 2 (MDM2) protein [21]. MDM2 acts as an E3 ligase for p53, promoting its polyubiquitination and subsequent degradation via the proteasome pathway [22]. The p53 protein plays a crucial role in promoting cell death by triggering cell cycle arrest and apoptosis in response to DNA damage. This mechanism is pivotal in eliminating cancer cells through drug treatment [23]. DUBs such as USP7, USP24, and USP10 have been extensively studied for their roles in the deubiquitination of p53 [24,25]. Particularly, USP7 deubiquitinates both p53 and MDM2, whereas USP24 stabilizes p53 stability through deubiquitination and promotes apoptosis upon cellular damage [26,27]. However, the role of USP19 in regulating p53 expression through DnaJC7 is not yet known. In this study, we identified DnaJC7 as a novel binding partner of USP19 and discovered that the USP19-DnaJC7 axis positively regulates p53 expression in cisplatin-treated EOC cells.

## 2. Materials and Methods

### 2.1. Single-Cell RNA Sequencing Analysis

Single-cell RNA sequencing (scRNA-seq) data were obtained from nine patients with high-grade serous carcinoma (HGSC) (GSE192898). Cells were considered low-quality and excluded if they met any of the following criteria: fewer than 200 or more than 10,000 detected genes per cell, fewer than 300 or more than 100,000 total RNA counts, or a mitochondrial gene proportion greater than 20%. Batch effects across samples were corrected using the Harmony R package (v1.2.4). Cell clustering was performed using the FindNeighbors and FindClusters functions in the Seurat R package (v5.4.0), with a resolution parameter set to 0.5 [28].

For each cell type, *p*-values and odds ratios for *USP19* expression were calculated using Fisher’s exact test. Differentially expressed genes (DEGs) between *USP19*-positive and *USP19*-negative epithelial cells were identified using the FindMarkers function in the Seurat R package with the following thresholds: log_2_ fold change ≥ 0.25, minimum expression in at least 10% of cells (min.pct = 0.1), and adjusted *p*-value < 0.05. Gene Ontology enrichment analysis was conducted using the g:Profiler web tool (version e113_eg59_p19_f6a03c19). Gene set enrichment scores were calculated at the single-cell level using the UCell R package (v2.2.1) with Hallmark gene sets [29]. Statistical comparisons between *USP19*-positive and *USP19*-negative epithelial cells were performed using the Wilcoxon rank-sum test.

### 2.2. Protein–Protein Interaction Network

We identified 122 putative binding partners of USP19 using the Integrated Interactions Database (IID; http://iid.ophid.utoronto.ca; accessed on 31 May 2025) and BioGRID (RRID: SCR_006334; https://thebiogrid.org; version 3.5.175; Table S1). A subnetwork of DnaJC7 and USP19 was depicted using Cytoscape (RRID: SCR_003032; https://cytoscape.org; version 3.9.0) based on the STRING network (RRID: SCR_005223; version 12.0).

### 2.3. Construction of Plasmid Vectors and Small Interfering RNA (siRNA)

*USP19*, subcloned into the pcDNA3.1-6-Myc vector, was used as described previously [14]. The *p53* gene was subcloned into the pcDNA3.1-3Myc vector (RRID: 176045), and the *MDM2* genes were subcloned into the pCS4-Flag vector. Myc-*p53* and Flag-*MDM2* were used, as described in a previous study [30]. The pOTB7-*DnaJC7* vector (hMU011596) was purchased from the Korea Human Gene Bank, Medical Genomics Research Center, KRIBB, Republic of Korea. *DnaJC7* was subcloned into the pCS4-Flag vector. Domain mutants of the *USP19* gene (∆1, ∆2, and ∆3) and the *DnaJC7* gene (∆1, ∆2, ∆3, and ∆4) were produced by subcloning. The Myc-*USP19* C506S gene was produced using site-directed mutagenesis PCR. The primers used for generating these domain mutants and Myc-*USP19* C506S form are listed in Table S2. Wild-type HA-*ubiquitin* and its mutants (K6, K11, K27, K29, K33, K48, K63, and M1) were used, as previously described [31]. Primers for site-directed mutagenesis PCR of *USP19* and domain mutants of *DnaJC7* and *USP19* are described in the Table S3. For the GST pull-down assay, *DnaJC7* was subcloned into the pGEX-4T vector. The siRNAs (GenePharma, Shanghai, China) for *DnaJC7* #1 (5′-AAG CAG UAC GAG ACU AUG AAA-3′), *DnaJC7* #2 (5′-AAG ACU CGC UAU GAC AGU GGA-3′) were evaluated for knockdown efficiency by Western blot analysis. Based on the results, siRNA#1 was selected for subsequent experiments (Figure S1). *USP19* (5′-GGA GGA GAU GGC AGU GGC A-3′) was used for knockdown experiments. The siRNA sequence targeting USP19 was validated in previous studies [14,32].

### 2.4. Cell Culture and Transfection

HEK293T (RRID: CVCL_0063; CRL-11268, ATCC, Manassas, VA, USA), HeLa (RRID: CVCL_0030; CCL-2, ATCC), TOV-112D (RRID: CVCL_3612, CRL-3593, ATCC), and SKOV3 (RRID: CVCL_0532; HTB-77, ATCC) cells were grown in Dulbecco’s Modified Eagles’ Medium (DMEM; 12800-017, Gibco, Grand Island, NY, USA) containing 10% fetal bovine serum (FBS; 12483-020, Gibco) and 1% antibiotic-antimycotic reagent (15240-062, Gibco). A2780 (RRID: CVCL_0134; T8089, Applied Biological Materials Inc., Richmond, BC, Canada) and OVCAR3 (RRID: CVCL_0465; HTB-161, ATCC) cells were grown in Roswell Park Memorial Institute (RPMI; 11875-093, Gibco) medium. All cells were maintained at 37 °C in a 5% CO_2_ atmosphere. For transfections, cells were treated with 10 mM polyethyleneimine (PEI; 6 µL/µg of DNA; 23966, Polysciences, Inc., Warrington, PA, USA) in 150 mM NaCl and Lipofectamine 2000 Reagent (11668-019, Invitrogen, Waltham, MA, USA) according to the manufacturer’s protocol. Cells were treated with cisplatin (PHR1624-200MG, Sigma-Aldrich, St. Louis, MO, USA) for 36 h (A2780 cells) and 48 h (HEK293T and HeLa cells).

### 2.5. Antibodies

Anti-Myc (RRID: AB_10637885; 1/200, 9E10) and anti-HA (1/500 to 1/3000, 12CA5) antibodies were obtained from hybridoma cell media. Anti-DnaJC7 (mouse, 5 µL/IP, 1/50 for ICC, sc-100716, Santa Cruz Biotechnology, Dallas, TX, USA; rabbit, 1/1000 to 1/3000, RRID: AB_2293179; 11090-1-AP, Proteintech Group, Rosemont, IL, USA), anti-USP19 (RRID: AB_2713918; 1/1000, 1/100 for ICC, 25768-1-AP, Proteintech Group), anti-Flag (1/3000 to 1/30,000, M185-3L, Sigma-Aldrich), anti-p53 (mouse, 3 µL/IP, sc-126, Santa Cruz Biotechnology; rabbit, 1/10,000 to 1/30,000, 10442-1-AP, Proteintech Group), anti-β-actin (1/5000, sc-4778, Santa Cruz Biotechnology), anti-Bax (1/10,000, 50599-2-Ig, Proteintech Group), anti-Bak (1/10,000, 29552-1-AP, Proteintech Group), anti-Caspase-3 (1/500 to 1/1000, 19677-1-AP, Proteintech Group), anti-Caspase-9 (1/1000, 10380-1-AP, Proteintech Group), and anti-p21 (1/3000, 10355-1-AP, Proteintech Group) antibodies were used for Western blotting (WB), ICC, and IP. For WB, the secondary antibodies used were mouse secondary antibody (1/30,000, 62-6820, Invitrogen), rabbit secondary antibody (1/10,000, sc-2357, Santa Cruz Biotechnology), and mouse TrueBlot (1/1000, 18-8817-30, ROCKLAND, Limerick, PA, USA).

### 2.6. Immunocytochemistry (ICC)

A2780, SKOV3, and OVCAR3 cells were seeded on glass coverslips in 12-well plates. The cells were briefly washed with phosphate-buffered saline (PBS; GBP0070, GeneSTAR, Seoul, Republic of Korea), fixed with 4% formaldehyde for 15 min, and washed again with PBS. For permeabilization, 0.2% Triton X-100 (T8787, Sigma-Aldrich) in PBS was used for 10 min, followed by another PBS wash. Cells were blocked with 1% bovine serum albumin (BSA; BSAS 0.1, Bovogen Biologicals, Keilor East, VIC, Australia) for 1 h at room temperature (20–22 °C) and then treated with primary antibodies (DnaJC7 and USP19) in 1% BSA at 4 °C. The cells were washed with 0.1% Tween 20 (4892, DUKSAN general science, Seoul, Republic of Korea) in PBS (PBStw) and incubated with Alexa-Fluor-568-conjugated goat anti-rabbit (a11011, Invitrogen) and Alexa-Fluor-488-conjugated goat anti-mouse (a11001, Invitrogen) at room temperature for 1 h. After washing with PBStw, the cells were stained with 4′,6-diamidino-2-phenylindole (DAPI; D9542, Sigma-Aldrich). The samples were visualized using a confocal microscope (Zeiss LSM880, Carl Zeiss Microscopy GmbH, Jena, Germany).

### 2.7. Western Blotting and Immunoprecipitation (IP)

WB was performed following the protocol described previously [33]. Harvested cells were lysed with lysis buffer (50 mM Tris-HCl [pH 7.5], 1 mM EDTA, 300 mM NaCl, 10% glycerol, and 1% Triton X-100), supplemented with a 1% protease inhibitor cocktail (P3100-001, GenDEPOT, Baker, TX, USA) and 1 mM phenylmethylsulfonyl fluoride (PMSF; P7626, Sigma-Aldrich). Samples were incubated on ice for 20 min, followed by centrifugation at 13,000 rpm at 4 °C for 20 min, and the supernatants were collected. The supernatants were boiled with 2X sodium dodecyl sulfate (SDS) buffer at 100 °C for 7 min, and then subjected to SDS-polyacrylamide gel electrophoresis. Proteins were transferred to a polyvinylidene difluoride membrane (IPVH00010, Millipore, Billerica, MA, USA). The membranes were blocked with 5% BSA or skim milk (232100, BD Bioscience, Franklin, NJ, USA) in TBST solution (20 mM Tris-HCl [pH 7.5], 150 mM NaCl, and 0.05% Tween 20) and incubated with primary antibodies (diluted in 2% BSA or skim milk and 0.02% sodium azide in TBST) at 4 °C overnight. After washing with TBST, the membranes were incubated with secondary antibodies diluted in 1% skim milk at room temperature for 1 h, followed by another wash. Protein bands were visualized using enhanced chemiluminescence solution (LF-QC0103, Young-In Frontier, Seoul, Republic of Korea). For the ubiquitination assay, cells were treated with 60 µM MG132 (F1100, UBPBio, Dallas, TX, USA) for 6 h prior to harvesting.

For IP, cell lysates were incubated with primary antibodies at 4 °C overnight on a rotator. Subsequently, protein A/G PLUS-Agarose Beads (sc-2003, Santa Cruz Biotechnology) were added and incubated for 2 h at 4 °C. Beads were washed with lysis buffer and then boiled with 2× SDS buffer at 100 °C for 7 min. The eluted proteins were analyzed by WB.

### 2.8. Glutathione S-Transferase (GST) Pull-Down Assay

For the GST pull-down assay, pGEX-4T (RRID: Addgene_73375, Addgene, Watertown, MA, USA) and pGEX-4T-DnaJC7 vectors were transformed into *BL21* cells. The cells were induced with 1 mM isopropyl β-D-1-thiogalactopyranoside (IPTG; V3955, Promega, Madison, WI, USA) at 18 °C for 18 h in Luria–Bertani broth (MB-L4488, KisanBio, Inc., Seoul, Republic of Korea) until reaching an A600 of 0.4. After sonication and centrifugation at 13,000 rpm for 20 min at 4 °C, the supernatants were incubated with Glutathione-Sepharose beads (17-0756-01, Cytiva, Marlborough, MA, USA) at 4 °C overnight on a rotator. The beads were washed twice with wash buffer (0.1 M Tris–HCl at pH 7.4, 0.5 M NaCl, and 20 mM imidazole at pH 7.4) and then incubated with HEK293T cell lysates overexpressing Myc-USP19, Myc-p53, or Flag-MDM2 at 4 °C overnight on a rotator. After two additional washes with wash buffer, the samples were boiled with 2× SDS buffer at 100 °C for 7 min and subjected to WB. GST-tagged proteins were visualized by staining with Coomassie Brilliant Blue R (SLBL7178V, Sigma-Aldrich) and G (SLBN7053V, Sigma-Aldrich) solutions.

### 2.9. Cycloheximide (CHX) Chase Assay

HeLa and A2780 cells were transfected with *siUSP19* for 24 h. Subsequently, the cells were treated with cycloheximide (100 µg/mL; 01810, Sigma-Aldrich). The cells were harvested at 0, 6, 12, and 24 h post-treatment, and protein stability was assessed using WB.

### 2.10. RT-qPCR and Primers

For RNA extraction, A2780 cells were lysed with TRIzol Reagent (15596018, Invitrogen) following the manufacturer’s instructions. cDNA was synthesized using the cDNA Synthesis Kit (CMRTK001, Cosmogenetech, Seoul, Republic of Korea) according to the manufacturer’s instructions. qPCR was performed using SYBR-Green PCR Master Mix (4367659, Applied Biosystems, Waltham, MA, USA) following the manufacturer’s instructions, and the amplicons were detected using a Real-Time PCR System (4376357, Applied Biosystems). Primers specific for *USP19* and *GAPDH* were employed as described in our previous study [34].

### 2.11. Cell Apoptosis Assay

The cell apoptosis assay was performed using flow cytometry (B53015, Beckman Coulter, Brea, CA, USA) and the Annexin V Apoptosis Detection Kit I (556547, BD Bioscience) following the manufacturer’s instructions. Transfected cells treated with cisplatin were washed with PBS, suspended in binding buffer, and stained with fluorescein isothiocyanate Annexin V and propidium iodide for 15 min at room temperature. Subsequently, the cells were analyzed using flow cytometry.

### 2.12. Cell Counting Kit-8 (CCK-8) Assay

For the CCK-8 assay, A2780 cells were transfected with siScramble, *siDnaJC7*, and *siUSP19* for 24 h. After transfection, cells were seeded into 96-well plates at a density of 1 × 10^5^ cells/well and treated with 0, 20, 40, and 60 µM cisplatin. After 24 h of treatment, cells were incubated with CCK-8 reagent (CK04, Dojindo, Kumamoto, Japan) in RPMI medium for 2 h. Optical density (OD) was measured at 450 nm using a microplate reader (Tecan Group Ltd., Männedorf, Switzerland).

### 2.13. Wound Healing Assay

For the wound healing assay, A2780 cells were seeded at a density of 1 × 10^6^ cells/well in 6-well plates and transfected with siScramble, *siDnaJC7*, or *siUSP19*. After 24 h, a scratch was made using a 10 µL micropipette tip, and the cells were treated with 60 µM cisplatin. Images were captured at 0, 18, and 36 h post-treatment. The wound width was measured using ImageJ software version 1.53k (RRID: SCR_003070; National Institutes of Health, Bethesda, MD, USA).

### 2.14. Transwell Assay

Transwell migration assays were performed using SPLInsert Hanging inserts with an 8 μm pore size (35206, SPL, Gyeonggi-do, Republic of Korea). A2780 cells were transfected with *siScramble*, *siDnaJC7*, or *siUSP19* for 24 h, and subsequently seeded into the upper chamber at a density of 4 × 10^5^ cells per well. After 12 h, cells were treated with 60 μM cisplatin. Following an additional 36 h incubation, non-migrated cells on the upper surface of the membrane were removed, and migrated cells on the lower surface were fixed and stained with crystal violet.

### 2.15. Statistical Analysis

Densitometric analysis was performed using ImageJ (RRID: SCR_003070; National Institutes of Health, Bethesda, MD, USA). Statistical analyses, including Student’s *t*-test and analysis of variance (ANOVA), were conducted using GraphPad Prism version 10 (RRID: SCR_002798; GraphPad Software, La Jolla, CA, USA). Significant *p*-values are indicated with asterisks (* *p*  <  0.05, ** *p*  <  0.01, and *** *p*  <  0.001). One-way or two-way ANOVA was followed by the Holm–Sidak multiple comparisons test as a *post hoc* test. Each experimental result shown represents the mean ± standard deviation (SD) of at least three independent experiments.

## 3. Results

### 3.1. scRNA-Seq Analysis in HGSC Patients

**Figure 1:**
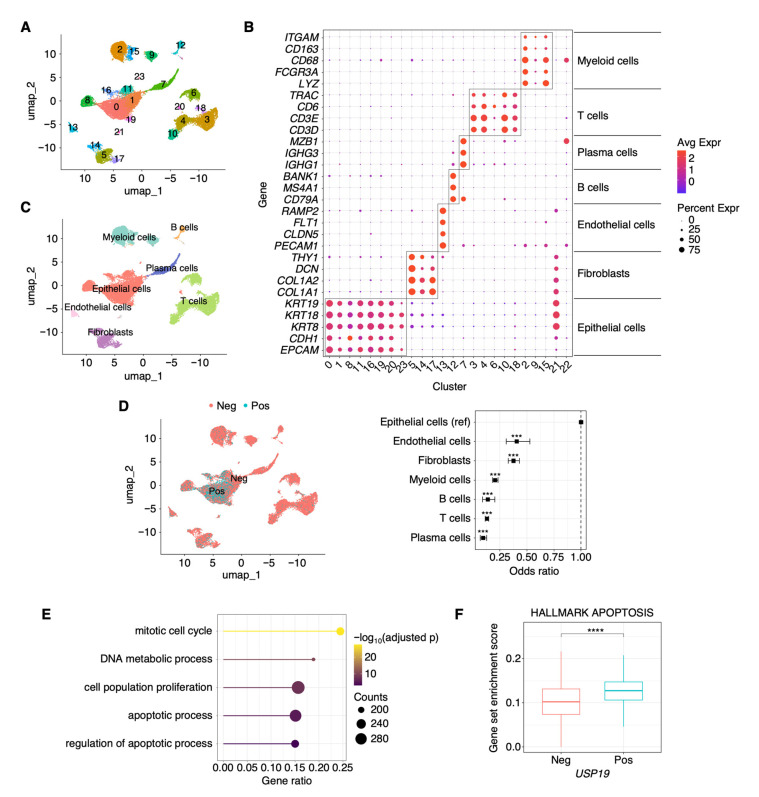
Association between *USP19* expression and epithelial cell-intrinsic programs in patients with HGSC. (**A**) UMAP visualization of scRNA-seq data shows cell clustering results. (**B**) Average expression of canonical marker genes across clusters. (**C**) UMAP visualization of cell type annotations. (**D**) Distribution of *USP19* expression across cell types. (**E**) Gene Ontology enrichment analysis based on DEG between *USP19*-positive and *USP19*-negative epithelial cells. (**F**) Comparison of Hallmark Apoptosis enrichment scores in epithelial cells according to *USP19* expression status. Neg, negative; Pos, positive. Significant *p*-values are indicated with asterisks (*** *p*  <  0.001 and **** *p*  <  0.0001).

To investigate which cell types predominantly express *USP19* and how *USP19* expression is associated with cellular characteristics, we analyzed scRNA-seq data from nine patients with HGSC. Clustering analysis identified a total of 23 distinct clusters comprising 25,730 cells (Figure 1A). To annotate cell types, the expression of canonical marker genes was systematically compared across clusters (Figure 1B,C). Clusters 21 and 22 were excluded from downstream analyses due to low cell numbers (n = 89 and n = 64) and ambiguous expression patterns.

When we examined *USP19* expression across the annotated cell types, epithelial cells displayed a higher proportion of *USP19*-expressing cells compared with other cell types (Figure 1D). To further characterize the transcriptional differences associated with *USP19* expression, we performed DEG analysis between *USP19*-positive and *USP19*-negative epithelial cells, followed by Gene Ontology enrichment analysis. This analysis revealed that apoptotic processes differed according to *USP19* expression status in epithelial cells (Figure 1E). Notably, the Hallmark Apoptosis gene signature was significantly enriched in *USP19*-positive epithelial cells (Figure 1F).

### 3.2. USP19 Binds to DnaJC7 and Co-Localizes with DnaJC7 In Vitro

**Figure 2:**
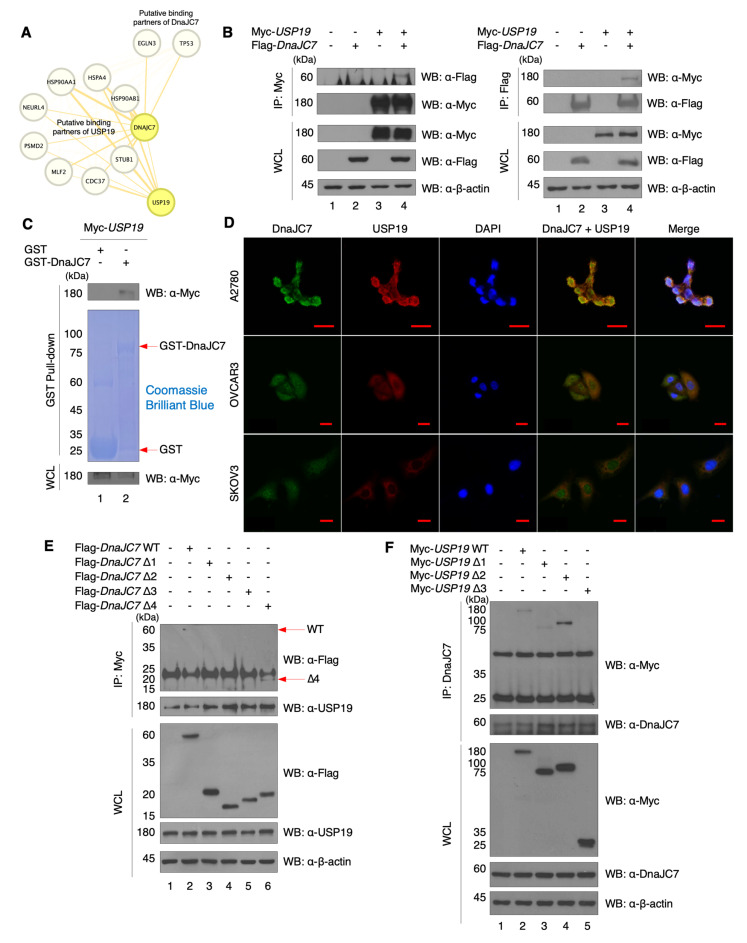
DnaJC7, a putative binding partner of USP19, binds with USP19. (**A**) The putative binding partner nodes of USP19 are connected to DnaJC7, and those of DnaJC7 are connected to p53. Both DnaJC7 and USP19 nodes are colored yellow. The visualization is generated using Cytoscape version 3.9 with STRING network, and the thickness of edges represents the interaction score between protein nodes. (**B**) Binding assays between USP19 and DnaJC7 are performed. Myc-USP19 and Flag-DnaJC7 are overexpressed in HEK293T cells, followed by IP using Flag and Myc antibodies, respectively. (**C**) GST pull-down assay is conducted to confirm the binding between DnaJC7 and USP19. Myc-USP19 overexpressed in HEK293T cells is incubated with purified GST-DnaJC7. (**D**) ICC assay is conducted to confirm the co-localization of DnaJC7 and USP19 in various EOC cell lines (A2780, OVCAR3, and SKOV3). A mouse-host DnaJC7 antibody and a rabbit-host USP19 antibody are utilized. 4′,6-diamidino-2-phenylindole DAPI is used to stain the nucleus. Scale bars represent 20 µm. (**E**) To determine which domain of DnaJC7 binds to USP19, domain mutant constructs of DnaJC7 are generated and a binding assay with Myc-USP19 is conducted in HEK293T cells. IP is performed using a Myc antibody, followed by detection using a USP19 antibody. (**F**) To confirm the binding between the domains of DnaJC7 and USP19, USP19 domain constructs are utilized in HEK293T cells. A mouse-host DnaJC7 antibody is used for IP, and a rabbit-host DnaJC7 antibody is used for WB.

Patients with high mRNA levels of *USP19* have a higher survival rate compared to those with low expression levels [6], and scRNA-seq analysis revealed that *USP19* expression is associated with enrichment of the apoptotic pathway in HGSC epithelial cells. To identify novel proteins regulated by USP19, we searched the BioGrid and Integrated Interactions Database (IID) PPI databases for putative binding partners. We identified 122 putative binding proteins for USP19 (Figure 2A and Table S1). We excluded proteins with a null score and selected those with anti-cancer effects when their expression was increased, based on research findings (Table S1). Consequently, DnaJC7, ACADSB, IRF3, and CRYL1 were identified as candidates. However, we prioritized DnaJC7 because it upregulates p53, a pivotal protein in cancer and the apoptosis pathway [21].

To check the binding between USP19 and DnaJC7 in vitro, we performed an IP assay in HEK293T cells, which are widely used for protein overexpression via exogenous DNA transfection [35]. Exogenously overexpressed Myc-USP19 and Flag-DnaJC7 were shown to bind each other (Figure 2B). Moreover, GST pull-down assay with purified GST-DnaJC7 confirmed their binding (Figure 2C). Co-localization analysis using an ICC assay with USP19 and DnaJC7 antibodies in A2780, SKOV3, and OVCAR3 cell lines revealed that DnaJC7 was localized in both the cytoplasm and nucleus, whereas USP19 is primarily located in the cytoplasm, adjacent to the nucleus, and possibly in the ER (Figure 2D). Lastly, we designed mutated domains of DnaJC7 and USP19 (Figure S2A,B) and found that USP19 binds to the J-domain of DnaJC7, whereas DnaJC7 interacts with the CS and USP domains in USP19 (Figure 2E,F).

### 3.3. USP19 Deubiquitinates DnaJC7 and Increases Its Protein Stability

**Figure 3:**
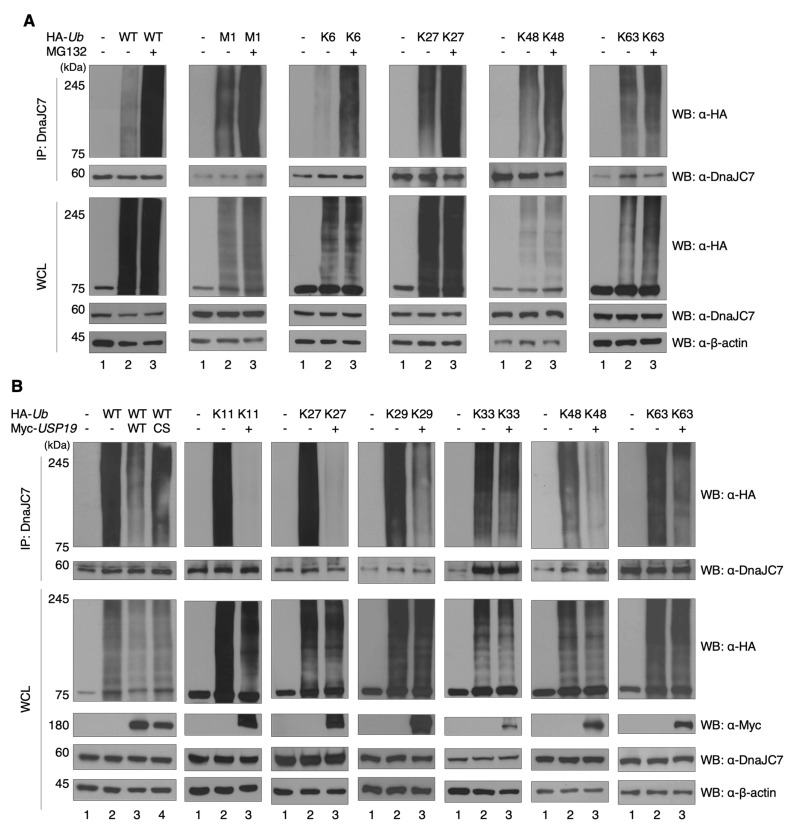
DnaJC7 is ubiquitinated and subsequently deubiquitinated by USP19. (**A**) Ubiquitination assay conducted in HEK293T cell line using wild-type and mutated ubiquitin constructs (M1, K6, K27, K48, and K63) and MG132. Rabbit anti-DnaJC7 antibodies are used for WB, and mouse anti-DnaJC7 antibodies are used for IP. (**B**) Deubiquitination assay of DnaJC7 with Myc-USP19 or Myc-USP19 (C506S) in HEK293T cell line. Wild-type ubiquitin and mutated ubiquitin constructs (K11, K27, K29, K33, K48, and K63) are used. For IP, mouse anti-DnaJC7 antibodies are used, and for WB, rabbit anti-DnaJC7 antibodies are used.

We first examined the ubiquitination status of DnaJC7, as its polyubiquitination has not been previously studied. Ubiquitination assays are performed using wild-type and mutated ubiquitin constructs with endogenous DnaJC7 (Figure S3A). DnaJC7 was polyubiquitinated with chains comprising eight ubiquitin linkages (M1, K6, K11, K27, K29, K33, K48, and K63) [36]. To identify which of these are associated with proteasomal degradation, we treated the cells with MG132, a proteasome inhibitor [37]. MG132 treatment led to the accumulation of M1-, K6-, K27-, K48-, and K63-linked polyubiquitin chains on DnaJC7, indicating their potential involvement in proteasomal degradation (Figure 3A). The remaining three linkages (K11, K29, and K33) showed no such accumulation upon MG132 treatment (Figure S3B).

We next performed deubiquitination assays to determine which polyubiquitin chains of endogenous DnaJC7 are deubiquitinated by USP19. Overexpressed USP19 deubiquitinated polyubiquitin chains, whereas catalytically inactive mutant USP19 (C506S) did not. USP19 deubiquitinates K11-, K27-, K29-, K33-, K48-, and K63-linked polyubiquitin chains on DnaJC7 (Figure 3B), while M1- and K6-linked polyubiquitin chains were unaffected (Figure S3C). Among these eight linkages, the K11-, K29-, and K48-linked polyubiquitin chains, which are known to mediate UPS-dependent protein degradation [38]. Therefore, of these three, only the K48-linked polyubiquitin chain appeared to be strongly associated with proteasomal degradation, and its deubiquitination was influenced by USP19 (Figure 3A,B, and Table S3). Therefore, we suggest that USP19 increases the protein stability of DnaJC7 by deubiquitinating its K48-linked polyubiquitin chain.

**Figure 4:**
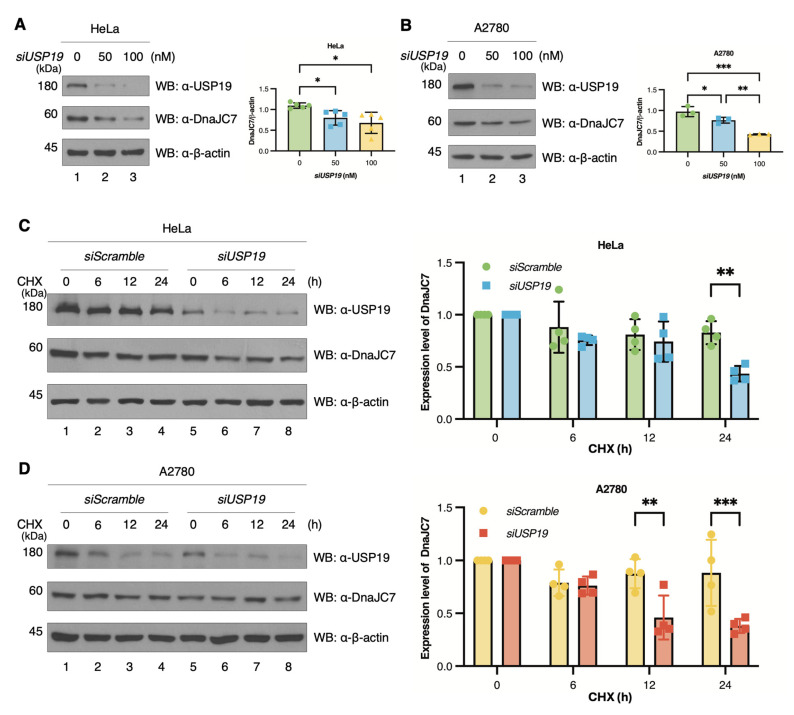
Knockdown of USP19 downregulates the protein stability and half-life of DnaJC7. (**A**) The stability of DnaJC7 is assessed using siRNAs targeting *USP19* in HeLa cells. The cells are treated with varying concentrations of *siUSP19*. The graph on the right displays the mean and standard deviation based on repeated measurements (n = 5) using one-way ANOVA. (**B**) A similar experiment is conducted using A2780 cells. The graph shows the mean and SD based on repeated measurements (n = 3) using one-way ANOVA. (**C**) To confirm that USP19 downregulates the half-life of DnaJC7, a CHX chase assay is performed. Following *siUSP19* transfection, the cells are treated with CHX and harvested at different time points. The graph on the right represents the fold change in DnaJC7 levels relative to the 0 h time point, showing mean and SD based on repeated experiments (n = 4) using two-way ANOVA. (**D**) The CHX chase assay is repeated using A2780 cells. The graph on the right represents the mean and SD from repeated experiments (n = 4) using two-way ANOVA. Significant *p*-values are indicated with asterisks (* *p* < 0.05, ** *p* < 0.01, and *** *p* < 0.001).

To verify this regulation, we knocked down *USP19* expression and detected its levels using an anti-USP19 antibody at 180 kDa [39]. USP19 knockdown resulted in decreased DnaJC7 expression levels (Figure 4A,B). HeLa cells have been shown to exhibit high levels of USP19 expression; therefore, the initial experiments were performed using them and employed *siUSP19* to confirm the expression of USP19 [40]. Subsequently, we repeated the experiment in A2780 cells, as previous research identified USP19 as a biomarker in ovarian cancer patients. Additionally, we found that knockdown of USP19 downregulates the half-life of DnaJC7 by inhibiting the synthesis of new protein upon treatment with CHX, an eukaryotic translation inhibitor [41]. The results showed that the half-life of DnaJC7 decreased upon USP19 downregulation (Figure 4C,D). These results indicate that USP19 positively regulates the protein stability of DnaJC7 via deubiquitination.

### 3.4. DnaJC7 Increases p53 Stability by Inhibiting Ubiquitination

**Figure 5:**
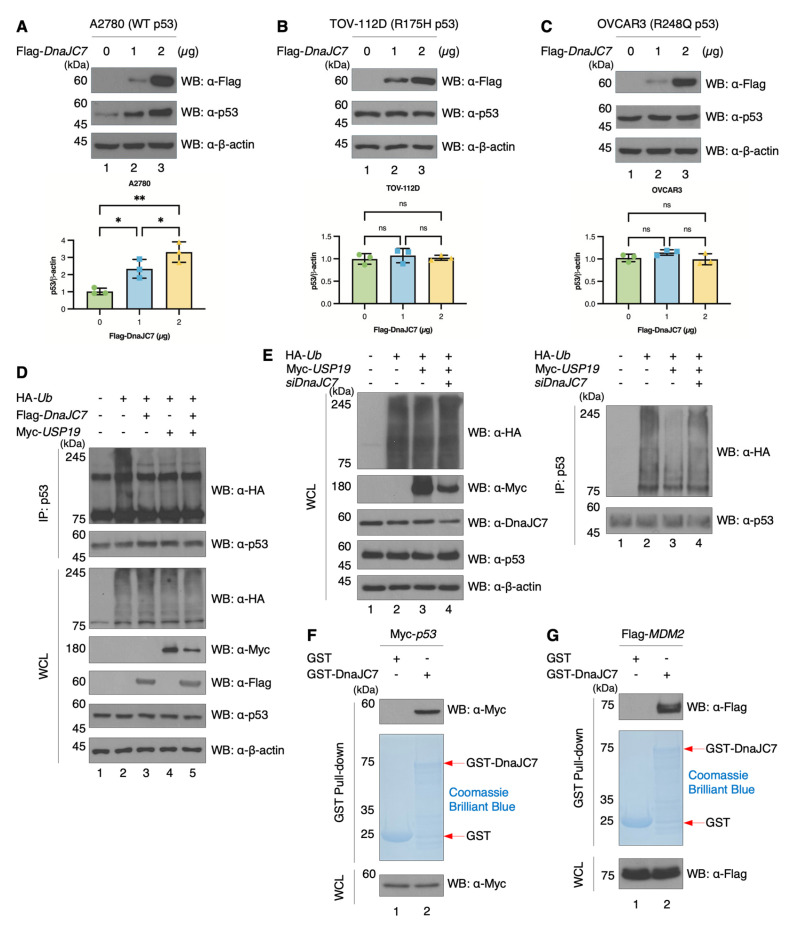
DnaJC7 upregulates the stability of p53 by inhibiting ubiquitination. (**A**–**C**) To confirm the association between DnaJC7 and the stability of p53 (WT, R175H, and R248Q), the expression of p53 is assessed in A2780 (WT), TOV-112D (R175H), and OVCAR3 (R248Q) cells overexpressing Flag-DnaJC7 in a dose-dependent manner. The graph on the right displays the mean and SD of p53 expression levels from repeated experiments (n = 3). Statistical analysis is conducted using one-way ANOVA. (**D**) To examine the polyubiquitination of p53 based on the expression levels of DnaJC7 and USP19, Flag-DnaJC7, Myc-USP19, and HA-Ub are overexpressed in HEK293T cells. Rabbit-host primary p53 antibodies are used for WB, whereas mouse-host primary p53 antibodies are employed for IP. (**E**) To identify the regulation of p53 ubiquitination through the USP19-DnaJC7 axis, Myc-*USP19* and *siDnaJC7* are used in HEK293T cells. (**F**) Binding of p53 and DnaJC7 is confirmed by incubating purified GST-DnaJC7 with HEK293T cell lysates overexpressing Myc-p53. (**G**) The binding of MDM2 and DnaJC7 is assessed by incubating purified GST-DnaJC7 with HEK293T cell lysates overexpressing Flag-MDM2. Significant *p*-values are indicated with asterisks (ns = not significant, * *p*  <  0.05, and ** *p*  <  0.01).

DnaJC7 is known to enhance p53 stability by disrupting its interaction with MDM2, an E3 ubiquitin ligase [21]. We examined whether DnaJC7 increases p53 levels and found that overexpression of DnaJC7 increased p53 stability in A2780 cells (Figure 5A). However, overexpression of DnaJC7 in TOV-112D (R175H) and OVCAR3 (R248Q) cells did not increase mutant p53 stability (Figure 5B,C). This is likely because p53 mutants such as R175H and R248Q induce structural changes in p53 [42,43,44]. Therefore, mutant p53 appears not to be regulated by DnaJC7 due to mutation-induced structural changes. Although DnaJC7 binds to both wild-type and mutant p53 (Figure S4A), it does not reduce the polyubiquitin chain of mutant p53 (Figure S4B).

Overexpression of either DnaJC7 or USP19 reduced p53 polyubiquitination, and DnaJC7 specifically decreased polyubiquitin chains on wild-type p53 (Figure 5D). However, co-overexpression did not produce a synergistic effect, suggesting that USP19 and DnaJC7 function within the same pathway. To further validate the role of DnaJC7 in this pathway, DnaJC7 was knocked down in the presence of USP19 overexpression, which restored p53 polyubiquitination levels. These results indicate that the USP19-DnaJC7 axis plays a critical role in regulating p53 stability (Figure 5E). GST pull-down assays further confirmed the interactions between DnaJC7 and both p53 and MDM2, suggesting that DnaJC7 can dissociate p53 from MDM2, thereby increasing p53 stability (Figure 5F,G). The interaction between USP19 and p53 was also confirmed (Figure S5).

### 3.5. The USP19-DnaJC7 Axis Maintains p53 Stability Under Cisplatin Treatment

**Figure 6:**
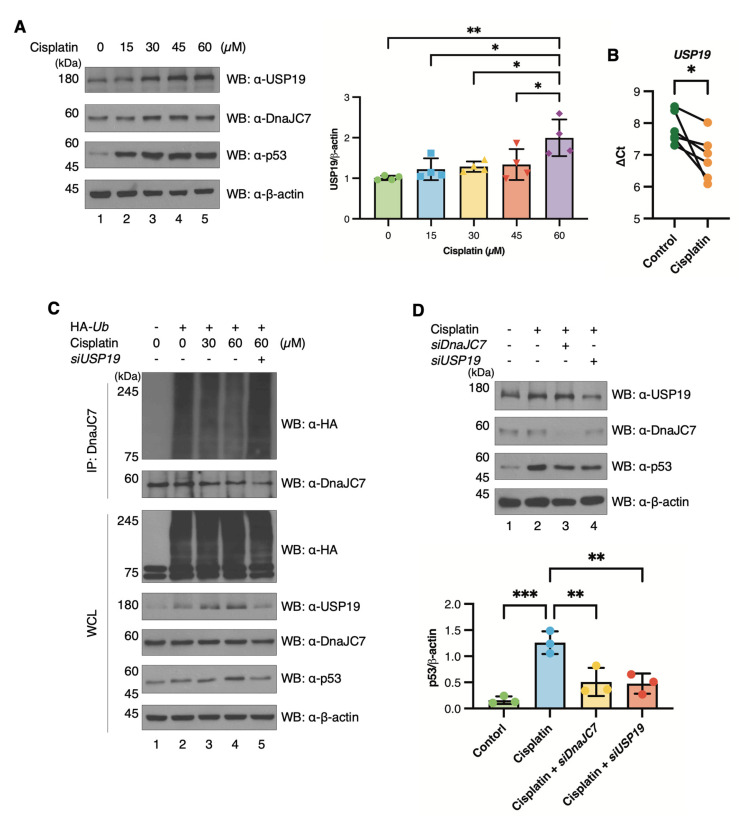
Cisplatin induces upregulation of USP19 and p53. (**A**) A2780 cells are subjected to cisplatin treatment in a dose-dependent manner to evaluate the expression of USP19, DnaJC7, and p53. The graph on the right represents data compiled from repeated experiments (n = 4), illustrating the mean and SD. Statistical analysis is conducted using one-way ANOVA. (**B**) RT-qPCR is performed on A2780 cells treated with cisplatin (60 µM for 36 h) and DMSO-treated cells. *GAPDH* serves as the housekeeping gene. The graph shows data from repeated experiments (n = 6), with statistical analysis conducted using an unpaired two-tailed Student’s *t*-test. (**C**) To investigate the polyubiquitination of DnaJC7 in response to cisplatin and USP19, A2780 cells are transfected with HA-*Ub*, allowed to grow, and then treated with cisplatin in a dose-dependent manner. Additionally, the cells are transfected with *siUSP19*. (**D**) To assess changes in p53 expression levels in response to USP19 and DnaJC7 expression during cisplatin treatment, A2780 cells are transfected with *siDnaJC7* and *siUSP19*. The cells are treated with cisplatin, and p53 expression levels are determined. The graph depicts data from repeated experiments (n = 3), with statistical analysis conducted using one-way ANOVA. Significant *p*-values are indicated with asterisks (* *p*   <  0.05, ** *p*   <  0.01, and *** *p*   <  0.001).

Cisplatin treatment has been shown to induce USP19 expression through ER stress and to upregulate p53 as part of the DNA damage response [45,46]. Consistent with this, p53 expression increased in a dose-dependent manner following cisplatin treatment, and USP19 levels also showed a moderate increase (Figure 6A). The cisplatin-induced upregulation of *USP19* was further supported by increased *USP19* mRNA levels detected by qPCR assays (Figure 6B and Figure S6A). We next investigated the intracellular localization of USP19 under cisplatin treatment, as USP19 has been reported to translocate into the nucleus upon γ-ray irradiation for regulating DNA damage repair [47]. Under normal conditions, USP19 was localized near the nucleus in the ER. Upon cisplatin treatment, USP19 translocated into the nucleus similarly to its behavior in response to γ-ray-induced DNA damage. Changes in the intracellular localization of USP19 were confirmed through ICC (Figure S6B,C).

Cisplatin-induced upregulation of USP19 enhanced its interaction with DnaJC7, as confirmed by endogenous IP (Figure S6C), and led to a decrease in DnaJC7 polyubiquitination (Figure 6C). In a ubiquitination assay for DnaJC7 using *siUSP19* with dose-dependent cisplatin treatment, high-dose cisplatin treatment reduced DnaJC7 polyubiquitin chain levels, and this effect was reversed upon treatment with *siUSP19* (Figure 6C). To determine whether p53 expression is affected by USP19 or DnaJC7 under cisplatin treatment, each protein was individually knocked down. While p53 expression increased with cisplatin treatment, this increase was abrogated upon knockdown of either DnaJC7 or USP19 (Figure 6D). Although USP19 and DnaJC7 are not primary drivers of p53 induction, they are required for maintaining p53 stability under cisplatin-induced stress conditions. Thus, p53 expression was highly influenced by the expression of USP19 and DnaJC7 under cisplatin treatment.

### 3.6. DnaJC7 and USP19 Are Required for Cisplatin-Induced Apoptosis

**Figure 7:**
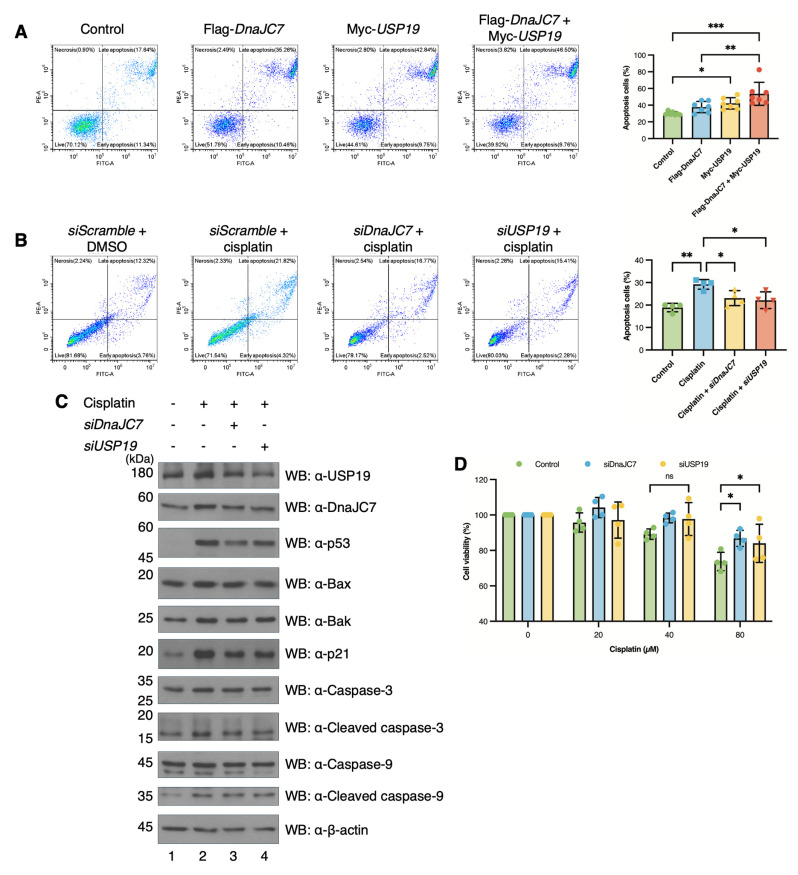
USP19 induces apoptosis through DnaJC7 and p53. (**A**) Apoptosis assays are conducted in A2780 cells via flow cytometry upon overexpression of Flag-DnaJC7 and Myc-USP19. The graph represents repeated experiments (n = 5), with statistical analysis performed using one-way ANOVA. (**B**) A2780 cells are transfected with *siDnaJC7* and *siUSP19*, followed by cisplatin treatment, and apoptosis is measured by flow cytometry. The graph is based on results of repeated experiments (n = 4), with statistical analysis performed using one-way ANOVA. Results are expressed as mean and SD. (**C**) A2780 cells transfected with *siDnaJC7* and *siUSP19* are treated with cisplatin, and the expression of components in the apoptosis signaling pathway is confirmed. (**D**) CCK-8 analysis was conducted in A2780 cells with cisplatin treatment and knockdown of DnaJC7 and USP19. The graph represents repeated experiments (n = 4). Two-way ANOVA is used for statistical analysis. Significant *p*-values are indicated with asterisks (ns = not significant, * *p*  <  0.05, ** *p*  <  0.01, and, *** *p*  <  0.001).

We examined whether DnaJC7 and USP19 promote apoptosis through p53, as both proteins positively regulate the stability of p53, a major mediator of apoptotic cell death. Overexpression of DnaJC7 and USP19 in A2780 cells exhibited higher proportions of apoptotic cells compared to the control (Figure 7A). While cisplatin treatment increased apoptosis, *siDnaJC7* or *siUSP19* in A2780 cells reduced the proportion of apoptotic cells to levels comparable to the untreated control group (Figure 7B).

We also investigated the expression levels of crucial proteins in the p53-induced apoptosis pathway. Cisplatin treatment increased the expression levels of BCL2-associated X (Bax), BCL2 antagonist/killer (Bak), caspase-3, caspase-9, cleaved caspase-3, cleaved caspase-9, and cyclin-dependent kinase inhibitor (p21), consistent with the p53 expression pattern. Knockdown of USP19 or DnaJC7 attenuated these (Figure 7C and Figure S7). We performed CCK analysis to determine cell viability following cisplatin treatment and knockdown of DnaJC7 and USP19. Cisplatin treatment reduced cell viability; however, knockdown of DnaJC7 and USP19 attenuated this reduction (Figure 7D).

## 4. Discussion

In this study, we identified DnaJC7 as a novel substrate of USP19 and demonstrated that USP19-mediated deubiquitination stabilizes DnaJC7 by preventing its proteasomal degradation. The stabilized DnaJC7 in turn disrupts the p53-MDM2 interaction, leading to p53 stabilization and activation of apoptotic signaling under cisplatin treatment. These findings provide a mechanistic basis for the association between elevated *USP19* mRNA expression and improved patient prognosis observed in our previous transcriptomic analysis. Here, we discuss three key aspects of these findings.

First, the role of USP19 in cancer appears to be context dependent. In EOC, elevated USP19 expression levels suppressed cancer progression, similar to observations in renal cell carcinoma and breast cancer [19,48]. In contrast, USP19 has been reported to promote cancer progression in colorectal carcinoma, cervical cancer, hepatocellular carcinoma, and gastric cancer [18,20,49,50]. One potential explanation for these divergent roles lies in the differential expression of USP19 isoforms. USP19 is typically categorized into two isoforms: one containing a transmembrane domain (TMD; USP19-TMD) anchored to the ER membrane, and the other ending with an EEVD (USP19-EEVD) amino acid sequence without the TMD at the C-terminus and localized in the cytoplasm [51]. In breast cancer cells, USP19-TMD inhibits epithelial–mesenchymal transition (EMT), whereas USP19-EEVD promotes EMT through transforming growth factor-β signaling [52]. Consistently, knockdown of USP19-TMD increased cell migration in our study (Figure S8), and endogenous ICC analysis confirmed that USP19 was localized proximal to the ER near the nucleus (Figure 2D). All experiments in this study were performed with the USP19-TMD isoform (Figure S2B). The elevated expression levels of the USP19-TMD isoform in EOC suppressed cancer progression, consistent with results in breast cancer. Therefore, understanding which USP19 isoforms are predominant in each cancer type is essential, and further studies are necessary to investigate the effects of the predominant USP19 isoform on each cancer and their relevance to patient prognosis.

Second, we utilized the A2780 cell line, which retains wild-type p53, to investigate the regulation of p53 by the USP19–DnaJC7 axis under cisplatin treatment. When DnaJC7 was overexpressed in two HGSC cell lines with mutant p53, it physically bound to mutant p53 but did not increase its protein levels (Figure 5B,C and Figure S4A). Given the diversity of p53 mutations and the limited scope of our investigation, we cannot draw general conclusions based on these two cell lines. However, this may be explained by mutation-induced structural changes in p53 (e.g., R175H and R248Q), which impair MDM2-mediated ubiquitination and degradation, rendering the DnaJC7-mediated regulatory mechanism largely ineffective. Consistently, ubiquitination assays confirmed that DnaJC7 does not reduce polyubiquitin chains of mutant p53 (Figure S4B).

These findings raise the question of how elevated USP19 expression is associated with improved prognosis and increased apoptotic signaling in HGSC, where TP53 mutations are prevalent. Notably, restoration of wild-type p53 activity has been shown to suppress tumor growth and induce apoptosis even in the presence of a co-existing p53 missense mutation in a mouse model, although without complete tumor regression [53]. This suggests that modulation of residual wild-type p53 may retain anti-tumor effects in tumors with heterozygous TP53 status. However, the extent to which the USP19-DnaJC7 axis contributes to this effect in TP53-mutant contexts remains unclear. Further studies using models with defined TP53 mutation status will be required to address this question.

**Figure 8:**
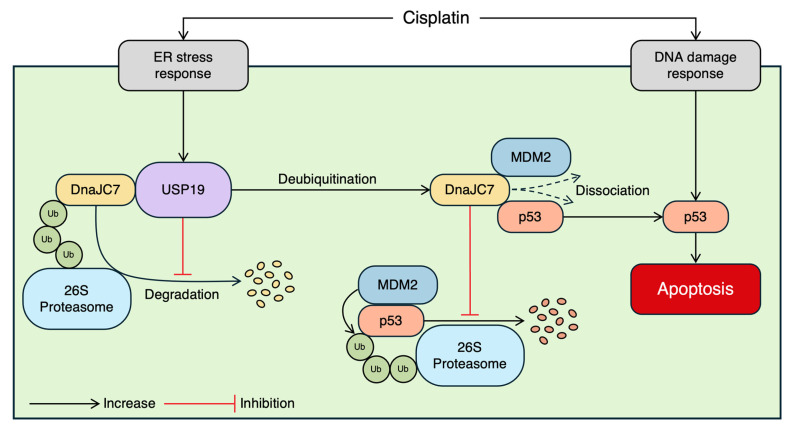
Schematic summary for USP19-DnaJC7-p53 axis. The schematic summary illustrates that cisplatin-induced ER stress and DNA damage responses upregulate USP19 and p53 expression levels, respectively. USP19 mediates the deubiquitination of DnaJC7, enhancing its stability and inhibiting its binding to p53 and MDM2. Consequently, this stabilization of p53 facilitates apoptosis triggered by cisplatin, which is orchestrated by the USP19–DnaJC7 axis.

Finally, based on our findings, we propose a mechanistic model in which cisplatin activates two parallel stress responses: an ER stress response that induces USP19 expression and a DNA damage response that induces p53 (Figure 8). USP19-mediated deubiquitination of DnaJC7 prevents its degradation by the 26S proteasome, thereby increasing DnaJC7 protein levels. The stabilized DnaJC7 then dissociates p53 from its negative regulator, ultimately enhancing p53 activity and apoptosis. From a therapeutic perspective, our findings suggest that enhancing the USP19-DnaJC7 axis may represent a promising strategy to stabilize wild-type p53 and improve the efficacy of platinum-based chemotherapy. One potential approach involves the development of deubiquitinase-targeting chimeras (DUBTACs) designed to facilitate the interaction between USP19 and DnaJC7, thereby promoting deubiquitination and preventing DnaJC7 degradation [54]. Consistent with this concept, previous studies have demonstrated that co-expression of wild-type and mutant p53 can modulate transcriptional activity in a context-dependent manner, highlighting the functional importance of residual wild-type p53 even in the presence of mutant forms [55]. Such strategies may stabilize wild-type p53 stability and strengthen apoptotic signaling in response to cellular stress.

In conclusion, we investigated the substrate positively regulated by USP19 and selected DnaJC7 for *in vitro* validation of their interaction. We confirmed the deubiquitination of DnaJC7 by USP19, leading to inhibit the degradation of DnaJC7 via the UPS. We identified an association between p53 and DnaJC7. The reduction in DnaJC7 and USP19 significantly affected p53 expression levels upon cisplatin treatment, suggesting their potential roles in reducing apoptotic cell death. The expression of USP19 is thought to affect patient prognosis in EOC because of this mechanism. Our study demonstrated that USP19 and DnaJC7 maintain p53 stability and enhance apoptotic cell death in EOC. This approach could advance the development of more effective cancer target therapies. Based on these findings, we propose that DnaJC7 and USP19 may serve as important targets for novel EOC treatment strategies.

## Data Availability

Publicly available scRNA-seq data were obtained from the GEO database at NCBI: https://www.ncbi.nlm.nih.gov/geo/ (accessed on 8 December 2025). Accession number is GSE192898.

## Supplementary Materials

The following supporting information can be downloaded at https://www.mdpi.com/article/10.3390/cells15100925/s1: Figure S1: A2780 cells were transfected with two independent siRNAs targeting DnaJC7 (#1 and #2). Knockdown efficiency was evaluated by Western blot analysis. Based on these results, siRNA #1 was selected for subsequent experiments; Figure S2: Schematic representation of domain mutations in DnaJC7 and USP19. (A) Diagram of DnaJC7 domain mutants. DnaJC7 contains nine tetratricopeptide repeat (TPR) domains and a J domain. (B) Diagram of USP19 domain mutants. USP19 consists of two CS domains: a ubiquitin-specific protease (USP) domain and a transmembrane domain (TMD); Figure S3: Ubiquitination and deubiquitination assays of DnaJC7 using specific ubiquitin mutants. (A) Schematic representation of the ubiquitin mutants used in the assays. In each construct, a single lysine residue is retained (indicated by its position), while all other lysine residues are mutated to arginine. (B) Ubiquitination assay conducted with K11-, K29-, and K33-linked ubiquitin mutants in the presence of MG132. (C) Deubiquitination assay of DnaJC7 performed with Myc-USP19 using M1- and K6-linked ubiquitin mutants; Figure S4: Ubiquitination assays of mutant p53 in the presence of DnaJC7. (A) Exogenous IP was performed using Flag-DnaJC7 and Myc-p53 (WT, R175H, and R248Q). (B) Ubiquitination assays were performed using Myc-p53 mutants (R175H and R248Q), WT ubiquitin, and Flag-DnaJC7; Figure S5: Interaction between p53 and USP19. Exogenous IP was performed using Flag-USP19 and Myc-p53, with an anti-Flag antibody used for IP; Figure S6: Interaction, intracellular localization of USP19 and DnaJC7, and USP19 expression following cisplatin treatment. (A) RT-qPCR analysis of USP19 expression following cisplatin treatment in A2780 cells, presented as 2^(−ΔΔCt) values (n = 5). (B) Immunocytochemistry (ICC) was conducted to examine the localization of USP19 and DnaJC7 following cisplatin treatment in A2780 cells. The scale bar represents 20 μm. (C) USP19 signal intensities in DMSO- and cisplatin-treated cells were quantified for whole-cell and nuclear regions in A2780 cells. Statistical analysis was performed using *t*-test (n = 12 and n = 7, respectively). (D) Endogenous IP was performed after cisplatin treatment (60 μM, 48 h). An anti-DnaJC7 mouse monoclonal antibody was used for IP, and a rabbit antibody was used for WB. Normal mouse IgG serves as a control. Significant *p*-values are indicated with asterisks (* *p*  <  0.05).; Figure S7: Expression of p53-mediated apoptosis pathway proteins. A2780 cells are treated with cisplatin and transfected with siDnaJC7 and siUSP19. Quantitative data represent independent experiments (n = 5), and statistical analysis was conducted using one-way ANOVA Significant *p*-values are indicated with asterisks (* *p*  <  0.05, ** *p*  <  0.01, and *** *p*  <  0.001); Figure S8: A2780 cells were treated with 60 µM cisplatin and transfected with *siDnaJC7* and *siUSP19* for the wound healing assay. The red lines indicate the wound border. In addition, transwell assays were performed with *siDnaJC7*, *siUSP19*, and cisplatin. Migrated cells were stained with crystal violet. The graph is based on replicate experiments (n = 5). Statistical analysis was performed using two-way ANOVA. Scale bars represent 20 μm. Significant *p*-values are indicated with asterisks (* *p*  <  0.05); Table S1: A total of 122 putative binding partners of USP19 were identified using the BioGrid (BD) and IID databases. Gene symbols represent each gene name. BioGrid scores are calculated based on high-throughput (HT) PPI data; Table S2: Primers used for generating the catalytically inactive mutant form of Myc-USP19 and domain mutants of Myc-USP19 and Flag-DnaJC7; Table S3: Summary of ubiquitination and deubiquitination of DnaJC7.
