# Supplementary material for: The USP19-DnaJC7 Axis Stabilizes p53 in Cisplatin-Treated Epithelial Ovarian Cancer
Source: Cells. 2026 May 18;15(10):925. doi: 10.3390/cells15100925 (PMC13204638; doi:10.3390/cells15100925)
Supplement: Supplementary file 1 [file cells-15-00925-s001.zip › cells-4240431-supplementary.pdf]

Supplemental information

The USP19–DnaJC7 axis stabilizes p53 in cisplatin-treated epithelial ovarian cancer

Yosuk Min[1], Donghyeon Kim[1], Hong-Beom Park[2], Hae-Seul Choi[3], Sohyun Hwang[1,3,*], and Kwang-Hyun Baek[1,2,4,*]

[1]Department of Life Science, CHA University, Gyeonggi-Do, Seongnam, 13488, Republic of Korea

[2]Department of Biomedical Science, CHA University, Gyeonggi-Do, Seongnam, 13488, Republic of Korea

[3]Department of Pathology, CHA Bundang Medical Center, CHA University School of Medicine, Gyeonggi-Do 13520, Seongnam, Republic of Korea

[4]Department of Bioconvergence, CHA University, Gyeonggi-Do, Seongnam, 13488, Republic of Korea

[*]Correspondence: baek@cha.ac.kr, Tel.: +82-31-881-7134; blissfulwin@cha.ac.kr, Tel.: +82-31-780-6019

This file contains the following contents:

Supplementary Figure S1
Supplementary Figure S2
Supplementary Figure S3
Supplementary Figure S4
Supplementary Figure S5
Supplementary Figure S6
Supplementary Figure S7
Supplementary Figure S8

Supplementary Table S1
Supplementary Table S2
Supplementary Table S3

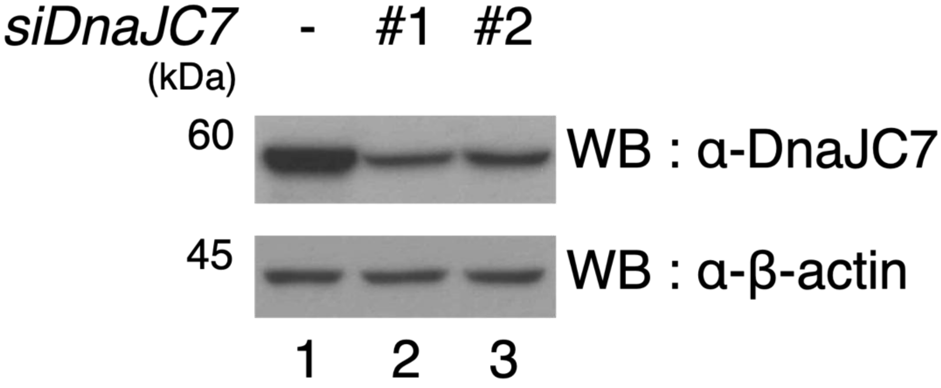

Figure S1. A2780 cells were transfected with two independent siRNAs targeting *DnaJC7* (#1 and #2). Knockdown efficiency was evaluated by Western blot analysis. Based on these results, siRNA #1 was selected for subsequent experiments.

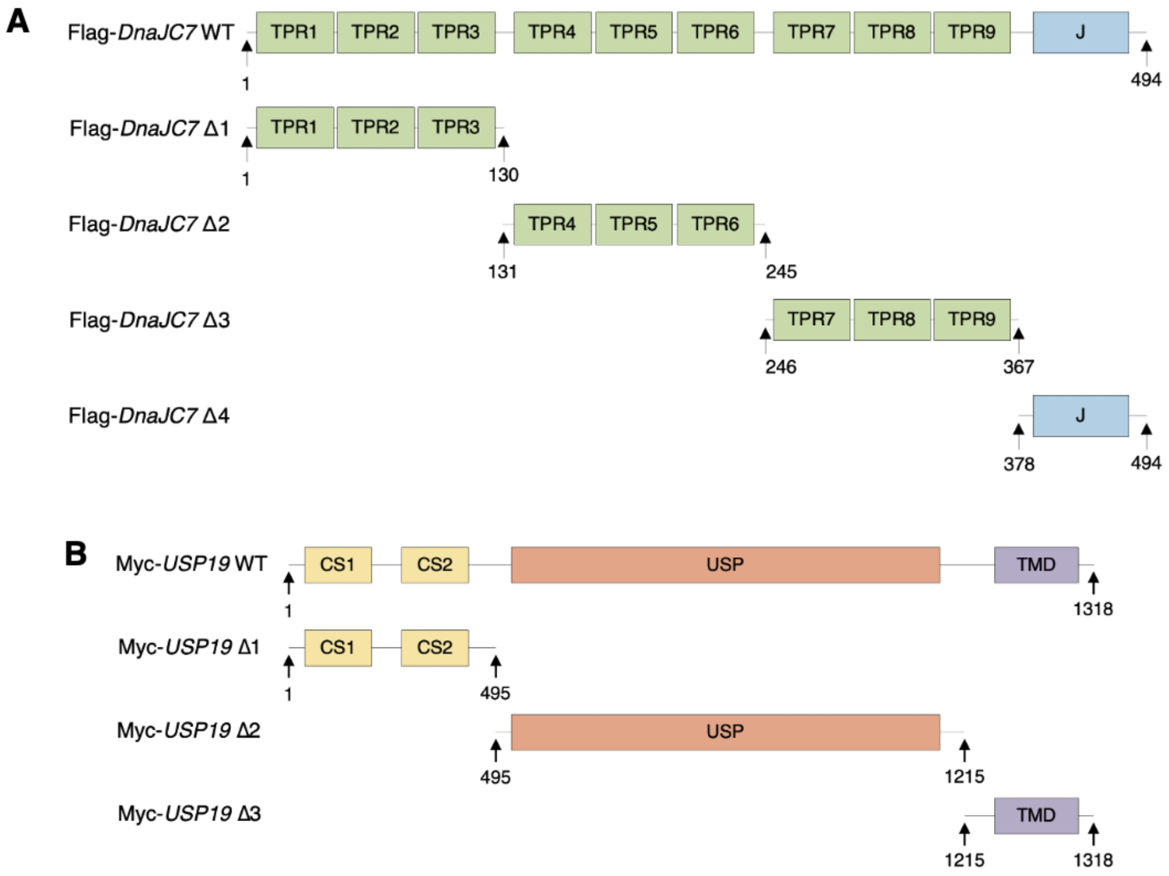

Figure S2. Schematic representation of domain mutations in DnaJC7 and USP19. (A) Diagram of DnaJC7 domain mutants. DnaJC7 contains nine tetratricopeptide repeat (TPR) domains and a J domain. (B) Diagram of USP19 domain mutants. USP19 consists of two CS domains: a ubiquitin-specific protease (USP) domain and a transmembrane domain (TMD).

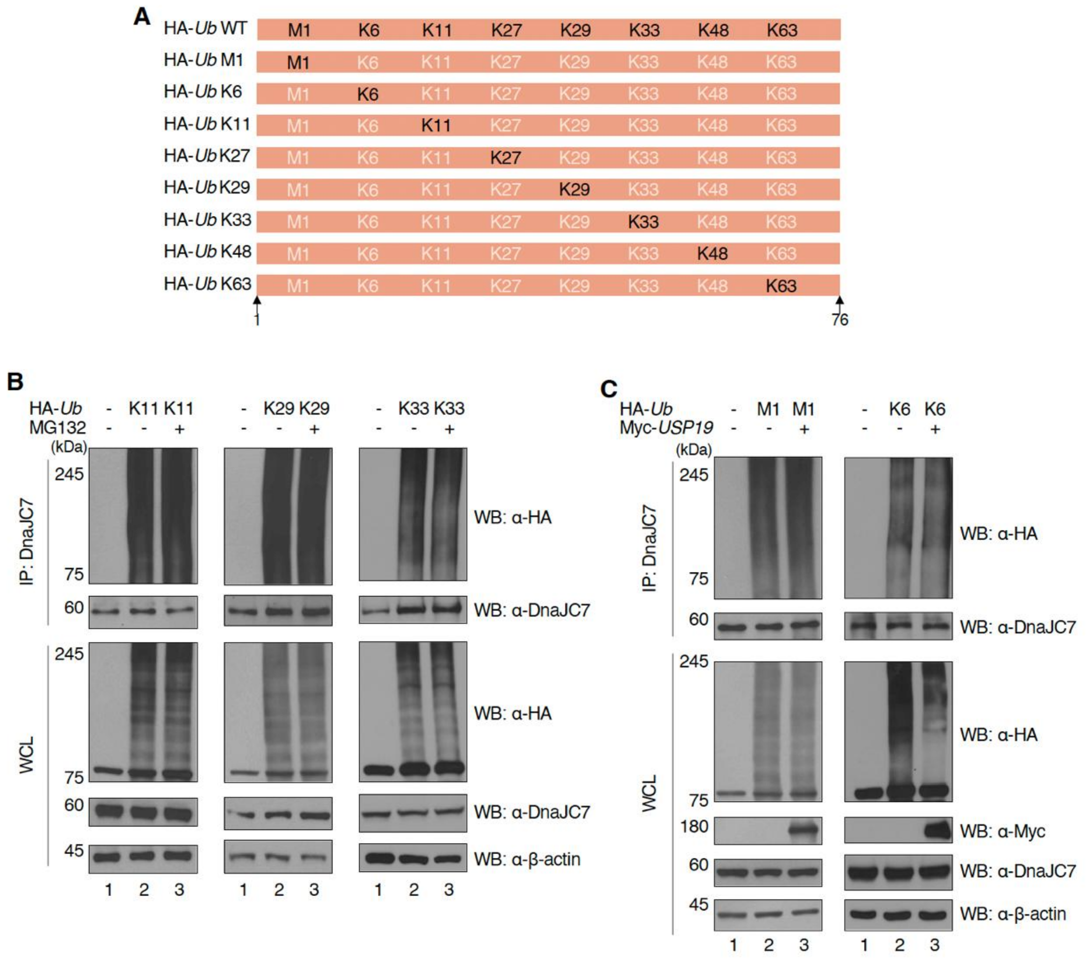

Figure S3. Ubiquitination and deubiquitination assays of DnaJC7 using specific ubiquitin mutants. (A) Schematic representation of the ubiquitin mutants used in the assays. In each construct, a single lysine residue is retained (indicated by its position), while all other lysine residues are mutated to arginine. (B) Ubiquitination assay conducted with K11-, K29-, and K33-linked ubiquitin mutants in the presence of MG132. (C) Deubiquitination assay of DnaJC7 performed with Myc-USP19 using M1- and K6-linked ubiquitin mutants.

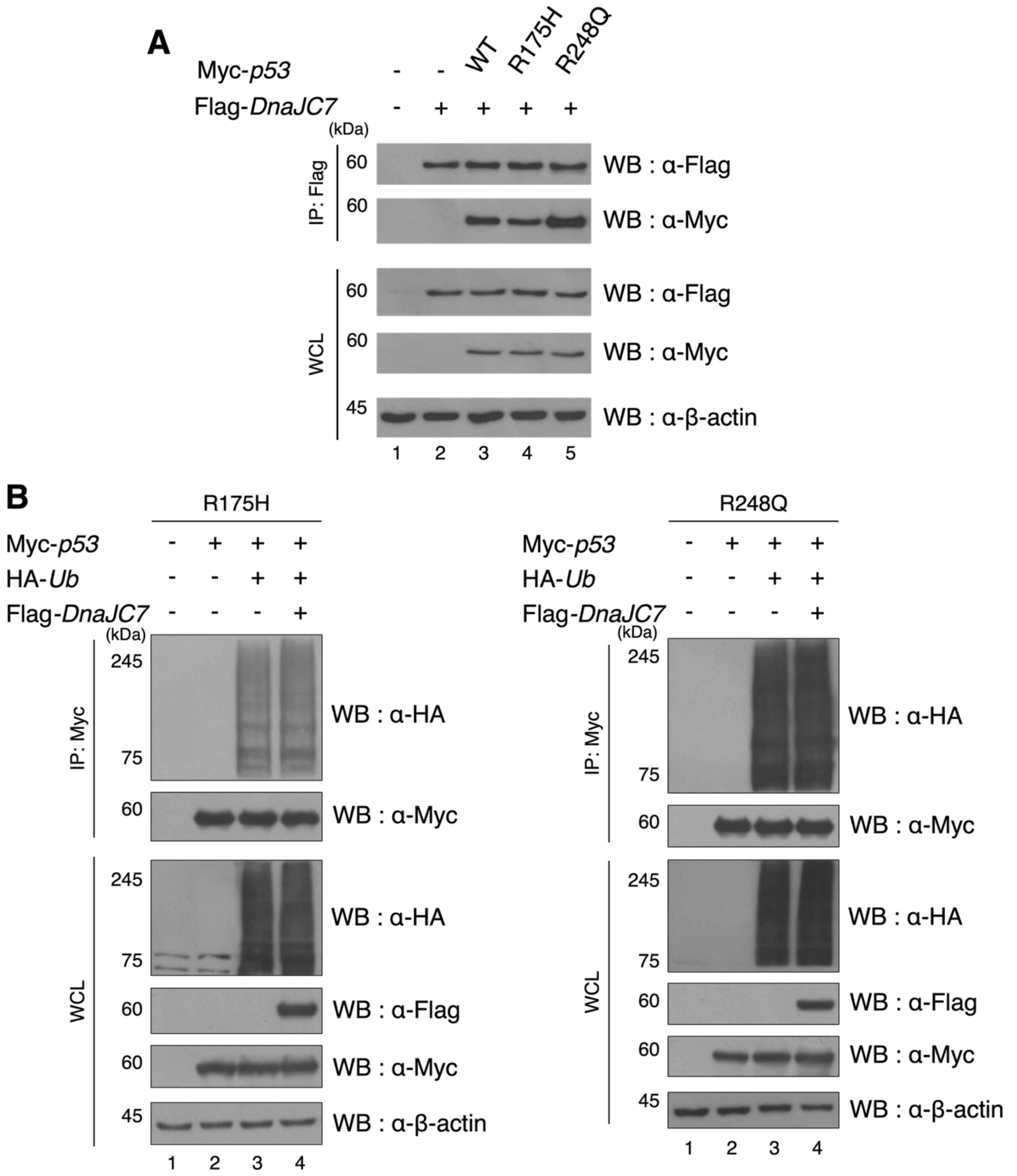

Figure S4. Ubiquitination assays of mutant p53 in the presence of DnaJC7. (A) Exogenous immunoprecipitation (IP) was performed using Flag-DnaJC7 and Myc-p53 (WT, R175H, and R248Q). (B) Ubiquitination assays were performed using Myc-p53 mutants (R175H and R248Q), WT ubiquitin, and Flag-DnaJC7.

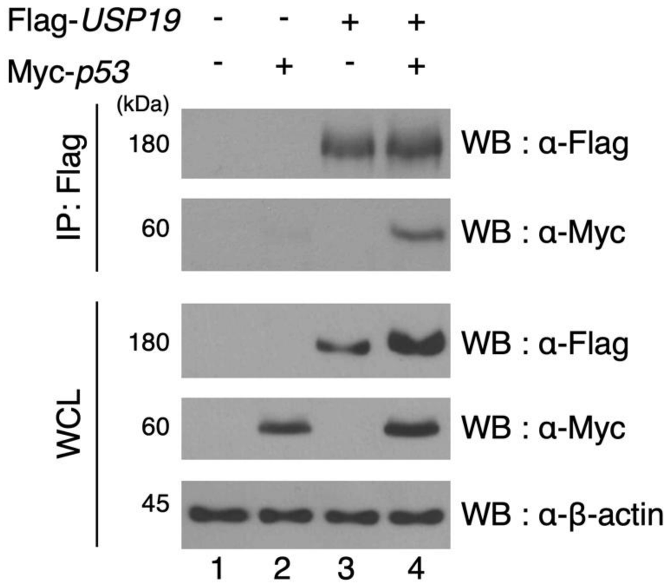

Figure S5. Interaction between p53 and USP19. Exogenous IP was performed using Flag-USP19 and Myc-p53, with an anti-Flag antibody used for IP.

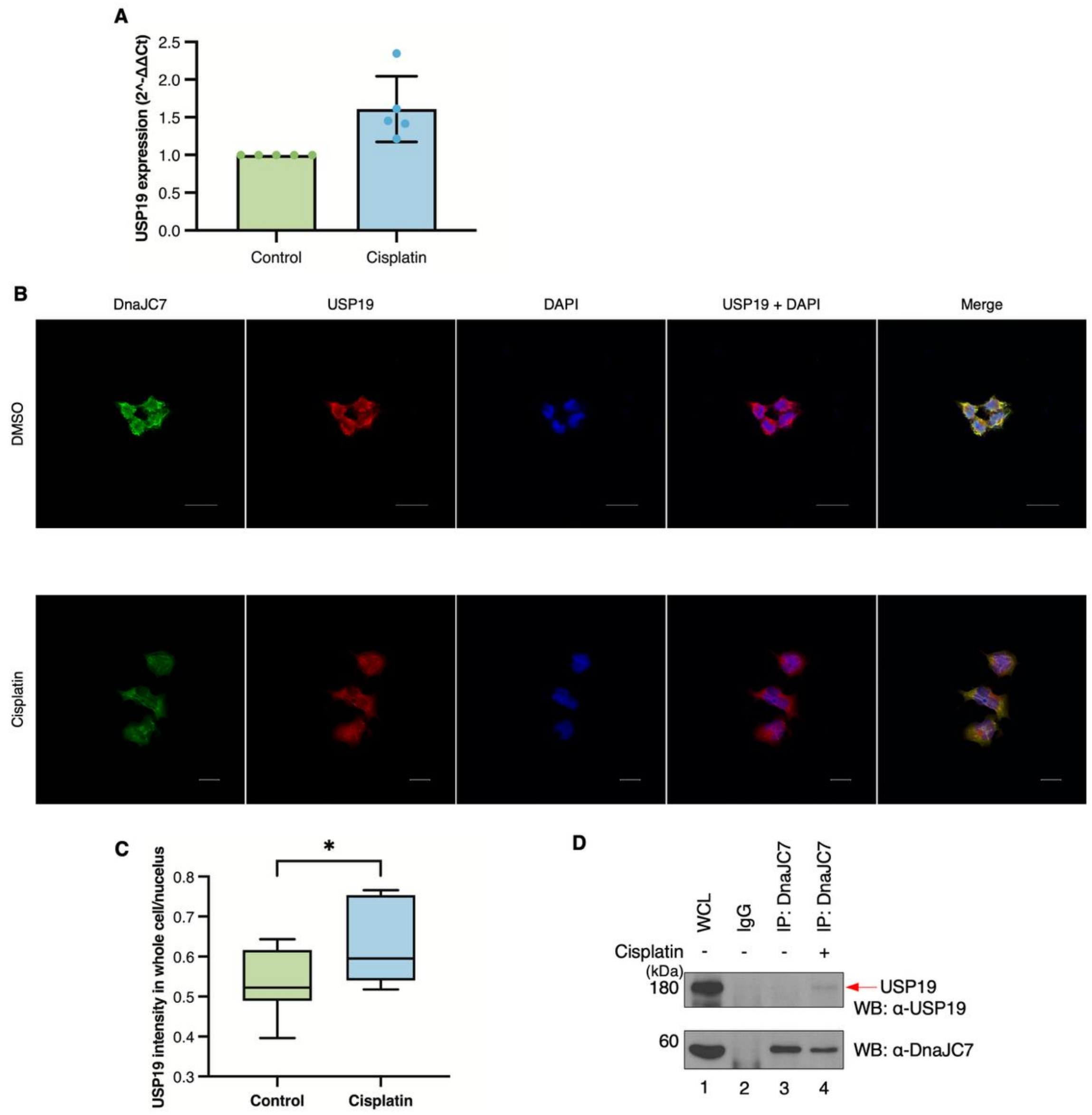

Figure S6. Interaction, intracellular localization of USP19 and DnaJC7, and *USP19* expression following cisplatin treatment. (A) RT-qPCR analysis of *USP19* expression following cisplatin treatment in A2780 cells, presented as 2^(-ΔΔCt) values (n = 5). (B) Immunocytochemistry (ICC) was conducted to examine the localization of USP19 and DnaJC7 following cisplatin treatment in A2780 cells. The scale bar represents 20 μm. (C) USP19 signal intensities in DMSO- and cisplatin-treated cells were quantified for whole-cell and nuclear regions in A2780 cells. Statistical analysis was performed using *t*-test (n = 12 and n = 7, respectively). (D) Endogenous IP was performed after cisplatin treatment (60 μM, 48 h). An anti-DnaJC7 mouse

monoclonal antibody was used for IP, and a rabbit antibody was used for Western blotting. Normal mouse IgG serves as a control. Significant *p*-values are indicated with asterisks (* $p < 0.05$).

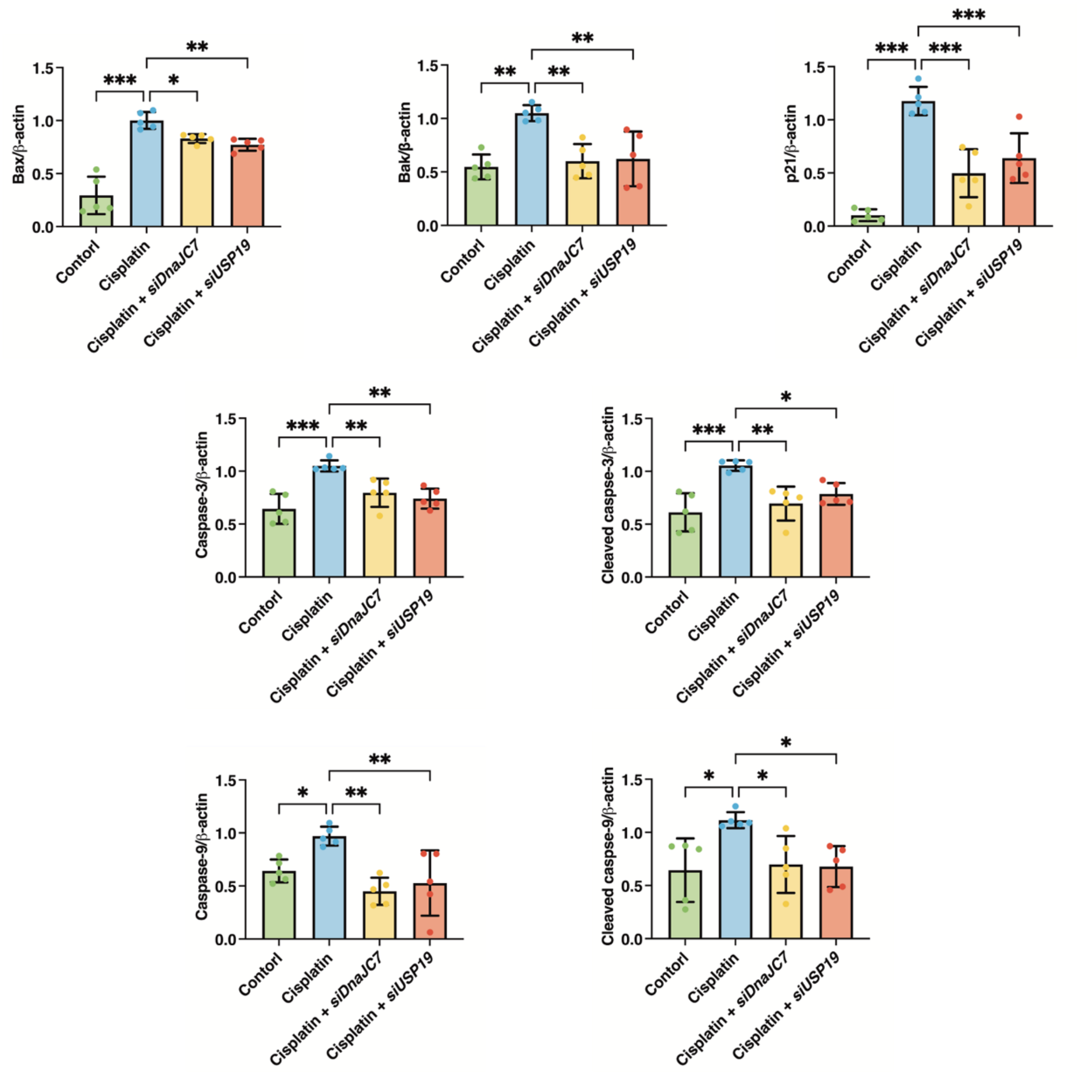

Figure S7. Expression of p53-mediated apoptosis pathway proteins. A2780 cells are treated with cisplatin and transfected with *siDnaJC7* and *siUSP19*. Quantitative data represent independent experiments (n = 5), and statistical analysis was conducted using one-way ANOVA. Significant *p*-values are indicated with asterisks (* $p < 0.05$, ** $p < 0.01$, and *** $p < 0.001$).

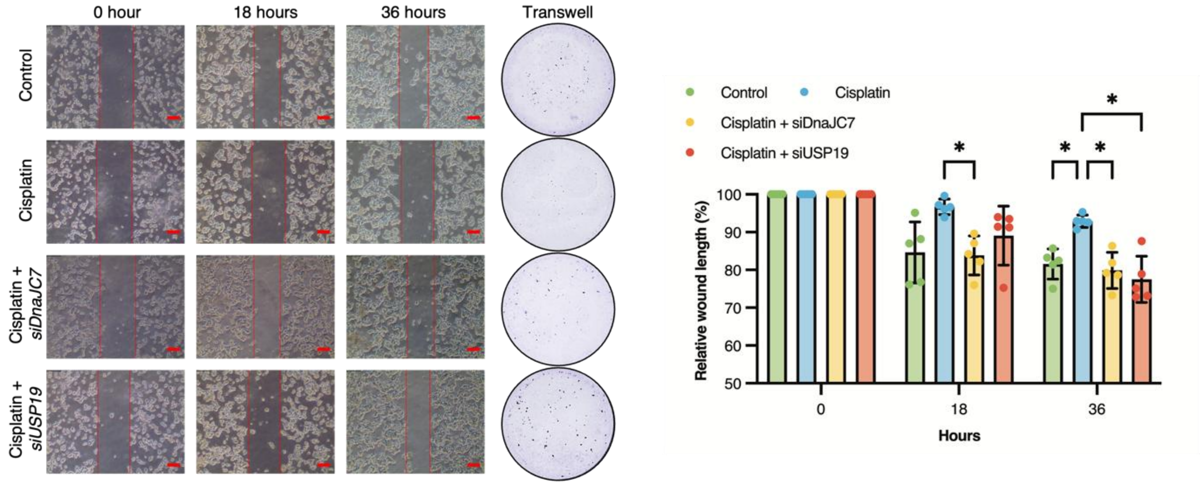

Figure S8. A2780 cells were treated with 60 μM cisplatin and transfected with *siDnaJC7* and *siUSP19* for wound healing assay. The red lines indicate the wound border. In addition, transwell assays were performed with *siDnaJC7*, *siUSP19*, and cisplatin. Migrated cells were stained with crystal violet. The graph is based on replicate experiments (n = 5). Statistical analysis was performed using two-way ANOVA. Scale bars represent 20 μm. Significant *p*-values are indicated with asterisks (* $p < 0.05$).

Table S1. A total of 122 putative binding partners of USP19 were identified using the BioGrid (BD) and IID databases. Gene symbols represent each gene name. BioGrid scores are calculated based on high-throughput (HT) PPI data.

Symbol	PPI database	BioGrid HT score	Role in cancer		Reference (PMID)	Reason not selected
ACADSB	BD	2.56	Tumor suppressing	Inhibit the proliferation by regulating cell ferroptosis	32776663	-
CRYL1	BD	0.96	Tumor suppressing	Promote apoptosis by PARP cleavage	19927314	-
DNAJC7	BD	0.78	Tumor suppressing	Regulate p53 stability	23261415	-
IRF3	BD	0.98	Tumor suppressing	Prevent colorectal tumorigenesis via inhibiting the nuclear translocation of β-catenin	33188184	-
ATG7	BD	0.94	Promoting cancer	Promote autophagy and tumor growth	38290972	To promote cancer
AURKC	BD	1.00	Promoting cancer	Induce proliferation in variety of cancers	39895780	To promote cancer
B4GALT2	BD	1.00	Promoting cancer	Oncogenic factor involved in immune exclusion	40010763	To promote cancer
BECN1	BD	Low throughput	Promoting cancer	Promote cancer progression	35422093	To promote cancer
BIRC2	IID	Low throughput	Promoting cancer	Increase breast tumor growth	32846130	To promote cancer
BIRC3	BD	Low throughput	Promoting cancer	BIRC3 up-regulation results in therapeutic resistance	26888114	To promote cancer
BIRC5	BD	Low throughput	Promoting cancer	Promote EMT and chemoresistance	38112021	To promote cancer
CACYBP	BD, IID	0.10	Promoting cancer	Promote proliferation	36576589	To promote cancer
CAMK2G	BD	5.12	Promoting cancer	Facilitate the adaptive redox homeostasis and drive cisplatin resistance	35039634	To promote cancer
CD2BP2	BD	Low throughput	Promoting cancer	CD2BP2 is crucial driver of breast cancer cell proliferation	39976088	To promote cancer
CDK1	BD	1.02	Promoting cancer	Lead to increased cell proliferation	32013835	To promote cancer
CKAP5	BD	Low throughput	Promoting cancer	Promote cell proliferation, migration, and invasion	35902921	To promote cancer
CSE1L	BD	1.03	Promoting cancer	Affect tumor progression	34659893	To promote cancer

CYP2W1	BD	0.76	Promoting cancer	Activated in colorectal cancer	27257736	To promote cancer
DDX60	BD	0.76	Promoting cancer	Promote pancreatic cancer cell growth	40950254	To promote cancer
DERL1	BD	Low throughput	Promoting cancer	DERL1 is associated with BRCA development	38858669	To promote cancer
DNM1L	BD	Low throughput	Promoting cancer	Promote GBM progression	39350223	To promote cancer
DOCK7	BD	1.81	Promoting cancer	Regulate a replication stress response that induces chemoresistance	33704464	To promote cancer
FKBP4	BD	Low throughput	Promoting cancer	Promote HCC development and glycolysis	39505995	To promote cancer
GCN1	BD	1.92	Promoting cancer	Promote HCC proliferation, migration, and invasion	40114124	To promote cancer
HERC2	BD	3.14	Promoting cancer	Promote inflammation-induced stemness and immune evasion in HCC	39538344	To promote cancer
HERC5	BD	Low Throughput	Promoting cancer	Promote breast cancer cell proliferation and migration	35671810	To promote cancer
HIF1A	BD	Low throughput	Promoting cancer	Promote metabolic reprogramming and progression of CRC	39227375	To promote cancer
HNRNPAB	BD	Low throughput	Promoting cancer	Play an important role in the malignant transformation of NSCLC	37153057	To promote cancer
HSP90AA1	BD	Low throughput	Promoting cancer	HSP90AA1 is critical factor in development of osteosarcoma chemoresistance	30153855	To promote cancer
HSPA4	BD	Low throughput	Promoting cancer	Induce immune evasion in gastric cancer	38589927	To promote cancer
HSPA8	BD	Low throughput	Promoting cancer	Development of refractory in colorectal cancer	37973552	To promote cancer
LAMTOR4	BD	Low throughput	Promoting cancer	Promote prostate cancer cell proliferation, migration, and invasion	39125671	To promote cancer
LRPPRC	BD	1.27	Promoting cancer	Promote resistance to CDK4/6 inhibition in lung cancer	37562037	To promote cancer
MAP3K7	BD	Low throughput	Promoting cancer	Promote chemoresistance	31300540	To promote cancer
ME1	BD	Low throughput	Promoting cancer	Promote gastric cancer growth and metastasis	29654155	To promote cancer

MGMT	BD	Low throughput	Promoting cancer	Regulate radioresponse in GBM, GSC, and melanoma	38811596	To promote cancer
MLF2	BD	Low throughput	Promoting cancer	Promote colorectal carcinogenesis	37438558	To promote cancer
MRPL42	BD	0.89	Promoting cancer	Promote glioma cell proliferation	29531015	To promote cancer
NXNL2	BD	Low throughput	Promoting cancer	Promote colon cancer proliferation and metastasis	37084033	To promote cancer
PIK3C3	BD	Low throughput	Promoting cancer	Promote breast cancer cell growth and metastasis	40372253	To promote cancer
PYGL	BD	2.17	Promoting cancer	Promote pancreatic ductal adenocarcinoma metastasis	37063425	To promote cancer
RNF2	BD	Low throughput	Promoting cancer	Promote the progression of colon cancer	34670117	To promote cancer
RORC	BD	Low throughput	Promoting cancer	Support pro-tumorigenic functions	39710149	To promote cancer
SAAL1	BD	1.81	Promoting cancer	Contribute to tumorigenesis and antitumor immunity mechanism	35963646	To promote cancer
SERBP1	BD	Low throughput	Promoting cancer	Promote development and progression of ovarian cancer	33526047	To promote cancer
SF3B3	BD	0.80	Promoting cancer	Promote colorectal cancer progression and metastasis	38671459	To promote cancer
SIAH1	BD	Low throughput	Promoting cancer	Promote epithelial mesenchymal transition of hepatocellular carcinoma	37038329	To promote cancer
SIAH2	BD	Low throughput	Promoting cancer	Play the vital role in tumorigenesis and cancer progression	34687788	To promote cancer
SKP2	BD	0.84	Promoting cancer	Associate with poor survival and adverse therapeutic outcomes	32014608	To promote cancer
SLC1A5	BD	1.02	Promoting cancer	SLC1A5 is a mitochondrial glutamine transporter for cancer metabolic reprogramming	31866442	To promote cancer
SNRNP200	BD	Low throughput	Promoting cancer	Inferior response to immunotherapy in TNBC	39285160	To promote cancer
SOHLH1	BD	0.92	Promoting cancer	Mediate glioma stem-like cell stemness and differentiation	40418209	To promote cancer

SQSTM1	BD	Low throughput	Promoting cancer	Decrease sensitivity to conventional chemotherapy	39971122	To promote cancer
SUCLG2	BD	2.09	Promoting cancer	Promote proliferation and tumorigenesis of LUAD cells	37904651	To promote cancer
SYVN1	BD	Low throughput	Promoting cancer	SYVN1 is related to tumor metastasis and growth	34196494	To promote cancer
TBK1	BD	Low throughput	Promoting cancer	Promote immune-evasion	36634707	To promote cancer
TGFBR1	BD	Low throughput	Promoting cancer	Associated with risk for several cancer	21461994	To promote cancer
TLN1	BD	0.80	Promoting cancer	Promote tumor cell invasiveness, proliferation, and metastatic progression	39589610	To promote cancer
TMEM33	BD	1.28	Promoting cancer	Regulate activation of SREBPs and lipid metabolism	34487377	To promote cancer
TRA	BD	Low throughput	Promoting cancer	Primary cancer tissue and positively correlated with chemoresistance and short survival	37327088	To promote cancer
TRAF6	BD	Low throughput	Promoting cancer	Promote breast cancer metastasis	36944688	To promote cancer
TRIM21	BD	1.2	Promoting cancer	Play cancer promoting and development of various cancers	36159815	To promote cancer
TRIM25	BD	Low throughput	Promoting cancer	Promote cell survival and growth of hepatocellular carcinoma	31953436	To promote cancer
UBC	BD, IID	Low throughput	Promoting cancer	Promote cell survival and proliferation	34966246	To promote cancer
ULK1	BD	Low throughput	Promoting cancer	Promote the progression of cervical cancer	39633065	To promote cancer
UNC45A	BD	2.22	Promoting cancer	UNC45A regulates cancer cell proliferation through ChK1 activation	25444911	To promote cancer
USP4	BD	Low throughput	Promoting cancer	Promote PI3K-mediated breast cancer metastasis	38134227	To promote cancer
USP7	BD	3.62	Promoting cancer	Promote cancer growth in blood, liver, breast, and kidney cancers	37775071	To promote cancer
VDAC1	BD	Low throughput	Promoting cancer	Regulate cell metabolism to provide advantage to cancer cell	39456237	To promote cancer

XIAP	BD	Low throughput	Promoting cancer	XIAP is known as anti-apoptotic protein	35661061	To promote cancer
XRN2	BD	0.78	Promoting cancer	XRN2 is required for cell motility and invasion in glioblastoma	35563787	To promote cancer
ZRANB1	BD	Low throughput	Promoting cancer	Promote breast cancer cell growth	29669287	To promote cancer
AIP	BD, IID	Low throughput	-	Suppress antiviral signaling and the induction of type I interferon	25911105	No cancer related role
ATAD1	BD	1.71	-	Exerted antiviral activity against HCV infection	37789674	No cancer related role
CCDC117	BD	0.91	-	Regulate DNA metabolism and proliferation	30742009	No cancer related role
CDC37	BD	Low throughput	-	Link to protein folding and autophagy	36520306	No cancer related role
CDC37L1	BD	Low throughput	-	Relative in tau regulation	33246057	No cancer related role
CFTR	BD	Low throughput	-	Cause cystic fibrosis	32326161	No cancer related role
DCTN3	BD	0.91	-	Aberrant expression of DCTN3 increases embryo aneuploidy	25645239	No cancer related role
DMWD	BD	4.97	-	Function as a cofactor that promotes USP12 enzymatic activity	33844468	No cancer related role
DOCK5	BD	0.90	-	DOCK4 deficiency is a critical to podocyte lipotoxicity	38161229	No cancer related role
ECEL1	BD, IID	Low throughput	-	Variant on the ECEL1 leading to neurodegenerative disorder	36794879	No cancer related role
EWSR1	BD	Low throughput	-	Tune germinal center response	37462917	No cancer related role
FAF2	BD	Low throughput	-	Reduce adipose triglyceride lipase lipolytic activity	39969435	No cancer related role
FERD3L	BD	1.00	-	FERD3L is involved in plaque vulnerability	26621503	No cancer related role
FKBP6	BD, IID	1.00	-	Suppress HCV replication	26567527	No cancer related role

FKBP8	BD	1.57	-	Modulate early steps of autophagosome formation	35090967	No cancer related role
FKBPL	BD	Low throughput	-	Induce ER fragmentation and ER-phagy	39251576	No cancer related role
FLNA	BD, IID	Low throughput	-	Regulate neuronal maturation in the developing cortex	38852754	No cancer related role
FUNDC1	BD	Low throughput	-	Promote hepatocyte injury	36828120	No cancer related role
HDAC1	BD	Low throughput	-	Control stem cell proliferation and neuronal regeneration in the adult hippocampus	35095417	No cancer related role
HDAC2	BD	Low throughput	-	Promote neuronal differentiation in adult hippocampus	35095417	No cancer related role
HMOX2	BD	5.12	-	Contribute to high-altitude adaptation	26781569	No cancer related role
IARS1	BD	2.02	-	IARS is tRNA synthetase	27426735	No cancer related role
KPTN	BD	0.80	-	Regulate mTROC1-related disorders affecting brain structure	37437211	No cancer related role
LAMP2	BD	Low throughput	-	Regulate autophagy in the thymic epithelium and thymic stroma	35535798	No cancer related role
LAT	BD	5.12	-	LAT is a linker for T cell activation	11752630	No cancer related role
MAP1LC3B	BD	Low throughput	-	-	-	No cancer related role
MAVS	BD	Low throughput	-	Mediate antiviral signaling	34016972	No cancer related role
NEURL4	BD	3.41	-	Act as main mitochondrial ART enzyme	35157000	No cancer related role
NFE2L1	BD	Low throughput	-	Regulate neurodegenerative disease	38150994	No cancer related role
NMNAT2	BD	0.78	-	Pivotal mediator of injury-induced axonal degeneration	38275737	No cancer related role
NSUN5P1	BD	0.93	-	-	-	No cancer related role

NTRK1	BD	Low throughput	-	NTRK1 knockdown induces mouse hippocampal neuronal damage	37907480	No cancer related role
NUP160	BD	1.04	-	Promote progression of diabetic nephropathy	35785044	No cancer related role
PAH	BD	Low throughput	-	Mutations in the PAH gene lead to phenylketonuria	23457044	No cancer related role
PARK7	BD	2.56	-	Mutations in PARK7 lead to autosomal recessive Parkinson's disease	35046029	No cancer related role
PDHA1	BD	1.71	-	PDHA1 hyperacetylation and inactivation enhance lactate overproduction	37479690	No cancer related role
PPP2R5B	BD	0.98	-	Cause human overgrowth	25972378	No cancer related role
RFXANK	BD	0.77	-	Regulate RFX7-associated signaling pathway	31864703	No cancer related role
RPAP3	BD	Low throughput	-	RPAP3 is essential for the formation of all R2TP and R2TP-like co-chaperone complex	32384603	No cancer related role
SEC61A1	BD	1.21	-	SEC61A1 is associated with autosomal dominant polycystic liver disease	36478640	No cancer related role
SEC61A2	BD	1.02	-	Maintain blood glucose homeostasis	39325584	No cancer related role
SEL1L	BD	Low throughput	-	SEL1L acts as checkpoint for preserving the survival and homeostasis for peripheral T cells	37644166	No cancer related role
SRPRB	BD	1.71	-	SRPRB is associated with variation in blood alcohol and acetaldehyde concentration	31002879	No cancer related role
STUB1	BD	Low throughput	-	Induce TFEB-induced autophagy-lysosome pathway	28754656	No cancer related role
TICAM1	BD	Low throughput	-	Regulate TLR3 activation	22205631	No cancer related role
TNPO3	BD	5.12	-	TNPO3 is an importin regulated by the small GTPase Ran	23878195	No cancer related role
TTC9C	BD	0.98	-	-	-	No cancer related role
HSP90AB1	IID	-	-	-	-	No HT score

OTULINL	IID	-	-	-	-	No HT score
RIGI	IID	-	-	-	-	No HT score

PMID is PubMed database identifier. ‘-‘ indicates no information

Table S2. Primers used for generating the catalytically inactive mutant of Myc-USP19 and domain mutants of Myc-USP19 and Flag-DnaJC7.

Genes		Primer sequences
Myc-*USP19* C506S	Forward	5'-GGC AAC ACC TCC TTC ATG AAC AGC-3'
	Reverse	5'-GCT GTT CAT GAA GGA GGT GTT GCC-3'
Flag-*DnaJC7* Δ1 (1-130)	Forward	5'-GAA TTC AAA TGG CGG CTG CCG-3'
	Reverse	5'-CTC GAG CTA AGC ATT TTT ATG ATC CAG-3'
Flag-*DnaJC7* Δ2 (131-245)	Forward	5'-GAA TTC AAC AGG CAC AAC AAG AGT TC3-'
	Reverse	5-'CTC GAG TCA CTT CTC GTG GTC A-3'
Flag-*DnaJC7* Δ3 (246-367)	Forward	5'-GAA TTC AAG CCT GCA TTG CCT GC-3'
	Reverse	5'-CTC GAG TTA TAG GAG CTG TTT GTG-3'
Flag-*DnaJC7* Δ4 (368-494)	Forward	5'-GAA TTC GGA AAA ATG CGC AGC TGG-3'
	Reverse	5'-CTC GAG CCT TAG CCA AAT TGA AAA AAG-3'
Myc-*USP19* Δ1 (1-494)	Forward	5'-GAA TTC TGA TGT CTG GCG GGG CC-3'
	Reverse	5'-CTC GAG TCA TGG CAG ACA CAC CTT-3'
Myc-*USP19* Δ2 (494-1217)	Forward	5'-GAA TTC TGG GCT TCA CTG GCC TTG TC-3'
	Reverse	5'-CTC GAG CTA AGG AGA GTT CCG CCG-3'
Myc-*USP19* Δ3 (1218-1318)	Forward	5'-GAA TTC TGG TGG AGA GGC CCC-3'
	Reverse	5'-CTC GAG TCA TCT CCA GCG ACT-3'

Table S3. Summary of ubiquitination and deubiquitination of DnaJC7.

Residues	DnaJC7 polyubiquitinated	Proteasomal degradation of DnaJC7	Deubiquitination of DnaJC7 by USP19	Proteasomal degradation by UPS
M1	O	O	-	-
K6	O	O	-	-
K11	O	-	O	O
K27	O	O	O	-
K29	O	-	O	O
K33	O	-	O	-
K48	O	O	O	O
K63	O	O	O	-

'-' indicates that DnaJC7 is not regulated by UPS or USP19 and 'O' indicates that DnaJC7 is regulated.
